# Papaya *CpbHLH1/2* regulate carotenoid biosynthesis-related genes during papaya fruit ripening

**DOI:** 10.1038/s41438-019-0162-2

**Published:** 2019-06-22

**Authors:** Dong Zhou, Yanhong Shen, Ping Zhou, Mahpara Fatima, Jishan Lin, Jingjing Yue, Xingtan Zhang, Li-Yu Chen, Ray Ming

**Affiliations:** 1 College of Resources and Environment, Fujian Agriculture and Forestry University, Fuzhou, Fujian 350002 China; 2 FAFU and UIUC-SIB Joint Center for Genomics and Biotechnology, Fujian Provincial Key Laboratory of Haixia Applied Plant Systems Biology, Key Laboratory of Genetics, Breeding and Multiple Utilization of Crops, Ministry of Education, Fujian Agriculture and Forestry University, Fuzhou, Fujian 350002 China; 3 Department of Plant Biology, University of Illinois at Urbana-Champaign, Urbana, IL 61801 USA

**Keywords:** Plant morphogenesis, Transcriptional regulatory elements, Transcriptomics

## Abstract

The ripening of papaya is a physiological and metabolic process associated with accumulation of carotenoids, alternation of flesh color and flavor, which depending on genotype and external factors such as light and hormone. Transcription factors regulating carotenoid biosynthesis have not been analyzed during papaya fruit ripening. RNA-Seq experiments were implemented using different ripening stages of papaya fruit from two papaya varieties. *Cis*-elements in lycopene β-cyclase genes (*CpCYC-B* and *CpLCY-B*) were identified, and followed by genome-wide analysis to identify transcription factors binding to these *cis*-elements, resulting in the identification of *CpbHLH1* and *CpbHLH2*, two bHLH genes. The expressions of *CpbHLH1/2* were changed during fruit development, coupled with transcript increase of carotenoid biosynthesis-related genes including *CpCYC-B*, *CpLCY-B*, *CpPDS2*, *CpZDS*, *CpLCY-E*, and *CpCHY-B*. Yeast one-hybrid (Y1H) and transient expression assay revealed that *CpbHLH1/2* could bind to the promoters of *CpCYC-B* and *CpLCY-B*, and regulate their transcriptions. In response to strong light, the results of elevated expression of carotenoid biosynthesis-related genes and the changed expression of *CpbHLH1/2* indicated that *CpbHLH1/2* were involved in light-mediated mechanisms of regulating critical genes in the carotenoid biosynthesis pathway. Collectively, our findings demonstrated several TF family members participating in the regulation of carotenoid genes and proved that *CpbHLH1* and *CpbHLH2* individually regulated the transcription of lycopene β-cyclase genes (*CpCYC-B* and *CpLCY-B*). This study yielded novel findings on regulatory mechanism of carotenoid biosynthesis during papaya fruit ripening.

## Introduction

Carotenoids are important structural and functional pigments existed in all plants, which are important for photosynthesis, plant growth and development. Many fleshy fruits, including papaya and tomato, have a dramatic flesh color change resulted from synthesized carotenoids accumulating in chromoplast during fruit ripening. The content of lycopene and β-carotene would have a significant increase from color-break stage to mature fruit, respectively, in red and yellow-fleshed papaya and tomato. As an adaptive characteristic, animals can be attracted by the changed pigments and disperse the matured seeds which are therefore able to germinate^1,2^.

During fruit ripening, carotenoid biosynthesis pathway is regulated by an genetically programmed mechanism that involves phytohormones and environmental factors such as, light, water, and temperature^3^. In papaya carotenoid biosynthesis pathway, with phytoene synthase (PSY) functioned, phytoene is synthesized by condensing two molecules of geranylgeranyl diphosphate (GGPP) in the plastids. Combining the phytoene desaturase (PDS) with ζ-carotene desaturase (ZDS), phytoene is further converted into lycopene which is the precursor of carotene. In nature, carotenoids usually exist in *trans*-structure. Only *trans*-lycopene can be catalyzed by downstream cyclase^4^. ZISO and CRTSO are key isomerases catalyzing the transformation of carotenoid *cis*-structure to *trans*-structure in plants. And the catalytic process also requires the participation of auxiliary factor flavin adenine dinucleotide (FAD)^5,6^. Afterward, one pathway lead to α-carotene via lycopene ε- and β-cyclase and the other result in β-carotene though chromoplast lycopene beta cyclase (*CYC-B*) or chloroplast lycopene beta cyclase (*LCY-B*) and further to zeaxanthin and lutein (Supplement Fig. S1)^7,8^. Meanwhile, recent studies have defined some transcription mechanisms required for the carotenoid biosynthesis-related genes during plant development. MADS-RIN, SPL-CNR, and NAC-NOR have been identified as crucial transcription factors (TFs) for regulating tomato fruit ripening resulting from mutations in these TF genes which inhibit fruit ripening process, such as softening and color development^9,10^.

Ethylene related TFs (EIN3/EILs and ERFs) regulate fruit development via implementing a transcriptional domino effect associated with ethylene-responsive genes. Reduced expression of *SIAP2a* and *SIERF6* results in carotenoid accumulation and ethylene biosynthesis in tomato, suggesting that *SIAP2a* and *SIERF6* act as negative regulators for carotenogenesis^11–13^. However, in Arabidopsis, these ethylene-responsive factors such as ERFs and RAP2.2 promote carotenoid biosynthesis by binding to *PSY* promoter^14^. In NAC family, carotenoid accumulation and ethylene synthesis can be positively regulated since *SlNAC4* actively regulate expression of the ripening regulator RIN and cannot be induced by ethylene^15^. In Citrus, through directly binding to promoters, *CsMADS6* upregulates the expression of *PSY*, *PDS*, and *CCD1*, implying *CsMADS6* regulate the multi-targeted carotenogenesis^16^. Loss-of-function mutations in *RCP1* belonging to MYB family led to downregulation of all carotenoid biosynthetic genes, suggesting *RCP1* boosts carotenoid biosynthesis during flower development in *Mimulus lewisii*^17^. Many other important TF families, including HD-ZIP HOMEOBOX PROTEIN-1 (HB-1) and TAGL1, have been proven to play important roles in fruit development in many plant species^18,19^.

bHLH is a transcription factor family in fruit ripening because they can interact with environmental cues driving multiple aspects of downstream morphogenesis^20^. In high-pigment tomato mutants, such as *hp1* and *hp2*, exposing to stronger light, anthocyanin and carotenoid will be accumulated. Meanwhile, in Arabidopsis, homologs of *DDB1* and *DET1* genes of *hp1* and *hp2* mutants showed negative regulation in light-mediated signaling pathway^21,22^. During tomato development, constitutive downregulation of *PIF1* levels resulted in increased accumulation of carotenoids in the fruit because *PIF1* directly repressed *PSY* gene expression^23^. Other results also showed that *PIF1* and other *PIFs* promote *PSY* gene expression and carotenoid accumulation through responding to light signals during daily cycles of light and dark in mature Arabidopsis^24^.

Acting as transcriptional cofactors, bHLHs regulate target genes with other transcription regulators. Since bHLHs proteins are absent from an appropriate DNA-binding domain, they usually function as bHLH heterodimer^25^. *PAR1* and *HFR1* interact with *PIFs* and keep them from matching the target genes’ promoters^26–28^. In recent decades, papaya fruit flesh has gained increased attention from breeders and consumers. Due to carotenoids content and composition, flesh color and nutritional quality have become increasingly important for fruit crop improvment^29^. *CpCYC-B* and *CpLCY-B* are crucial genes controlling flesh color and carotenoids profile in papaya. The red color of papaya flesh is from accumulating lycopene, while the yellow color is attributed to lycopene conversion to β-carotene and β-cryptoxanthin. Studying the regulatory mechanisms of *CpCYC-B* and *CpLCY-B* could lead to potential applications for improving fruit color and quality.

To unravel the molecular mechanism of papaya flesh color, we examined how *bHLHs* regulate *CpCYC-B* and *CpLCY-B* during papaya fruit development. To delineate the function of *bHLHs* on *CpCYC-B* and *CpLCY-B*, we conducted genome-wide identification of TFs targeting carotenogenesis by RNA-seq assay coupled with *cis*-element analysis and isolation for narrowing the numbers of TF families regulating *CpCYC-B* and *CpLCY-B* genes. More than 15 TF families were identified and 8 TFs were selected that may have positive or negative role in regulating *CpCYC-B* and *CpLCY-B* during fruit ripening. Yeast one-hybrid experiments and dual-luciferase transient expression assays demonstrated *CpbHLH1* and *CpbHLH2* can directly bind to the promoter upstream regions of *CpCYC-B* and *CpLCY-B* and individually inhibit or promote their transcription. Furthermore, we demonstrated that light might also involve in the regulation of *CpbHLH1* and *CpbHLH2* during fruits ripening.

## Materials and Methods

### Plant material and treatment

Red-fleshed papaya (*Carica papaya* L., cv. Hongling, SunUp, AU9) fruits at green, color break, and ripening stages were collected from experimental stations in Anxi and Yangzhong in Fujian, China. Two post-harvest treatments have been subjected: dark and light. During dark and light treatment, fruits were kept at 28 °C for 2 days. Fruits with the same morphology were selected, such as shape, maturity, weight, and without virus defects. All experiments will be biologically replicated with three samples after being frozen with liquid nitrogen or −80 °C.

### RNA extraction, library construction, gene isolation, and sequence analysis

Through grinding frozen papaya flesh samples, total RNA was extracted from fruits according to RNA-prep pure Plant Kit (Huayueyang) protocol. The quality and concentration of total RNAs were checked on an Agilent 2100 Bioanalyzer. After matching the qualification, mRNA samples were synthesized as cDNA and further constructed into libraries according to NEBNext Ultra RNA library Pre Kit for Illumine (NEB, E7530). The cDNA libraries were sequenced using Illumina NovaSeq with paired-end 150nt read length. By analyzing the RNA-seq data, eight differentially expressed genes, named *CpNAC3; CpbZIP-1/2/3; CpERF1/2; CpbHLH-1/2* were identified from the database for different papaya-ripening stages.

### Quantitative real-time PCR analysis

The experiments of qRT-PCR were performed with above RNA libraries. The primers applying to qRT-PCR analysis were designed as shown in Supplementary Table S2. The resulting qRT-PCR data were computed and analyzed using the formula 2^−ΔΔCt^ ^30^. *CpTBP1* adopted as an internal standard in papaya^31^. All experiments were implemented with three biological replications. The final values were presented with the mean of three biological replications.

### DNA extraction and promoter isolation

Total genomic DNA of all samples was extracted according to the Plant DNA Isolation Reagent protocol (Takara). Genomic sequences in promoters of *CpZDS*, *CpPDS*, *CpLCY-E*, *CpCYC-B*, *CpLCY-B*, and *CpCHY-B* were amplified from papaya genomic DNA (ftp://ftp.jgipsf.org/pub/compgen/phytozome/v9.0/Cpapaya) (primers were listed in Supplementary Table S3).

### Construction of vectors and plant transformation

Deletion constructs of *CpCYC-B* and *CpLCY-B* 5′ promotor sequences were amplied based on the annotated papaya genome at positions −0.5, −1.0, −1.5 kb (including −0.2/−0.3/−0.4/−0.5-absent element) and cloned into pDNOR207 vector using Gateway technology (Invitrogen). The targeting promoter fragments were then sub-cloned into pMDC162 and pGWB633 vectors using the Gateway conversion system. The *Agrobacterium tumefaciens*-mediated transformation system was implemented to transform pMDC162 and pGWB633-deletion promoters’ constructs into Arabidopsis Columbia (Col-0) ecotype.

### Dual-luciferase transient expression assay

The transcription activity of *CpbHLH1* and *CpbHLH2* was analyzed through the double luciferase reporter gene system in tobacco (*N. benthamiana*). As effectors the CDS sequences of *CpbHLH1* and *CpbHLH2* were inserted into the pGreenII 62-SK vectors, including a CaMV35S-Rluc and internal reference REN regulated by the 35S promoters (pG62-*CpbHLH1* and pG62-*CpbHLH2*). Meanwhile, the promoters of *CpCYC-B* and *CpLCY-B* genes were cloned into the pGreenII 0800-LUC double-reporter vectors^32^. Through co-transforming the *Agrobacterium*, respectively, containing effector and reporter constructs into tobacco leaves, the luciferase activities of LUC and REN were obtained using luciferase assay kit (Promega). At least eight biological replications were performed. Luminoskan Ascent Microplate Luminometer (Thermo Fisher Scientific) was used to collect data.

### Yeast one-hybrid (Y1H) and yeast two-hybrid (Y2H) assays

Y1H experiments were completed according to the Matchmaker Gold Y1H System protocol (Clontech). The CDS sequence of *CpbHLH1* and *CpbHLH2* were cloned into pGADT7 to fuse with the activation domain in the vector. The promoter fragments and short repeat promoter elements of *CpCYC-B* and *CpLCY-B* were cloned into pHIS2 vector. Co-transforming PGADT7 vectors containing transcription factors and pHIS2 vectors embodying promoters into yeast strain Y187. Yeasts were cultivated on SD basic medium without Leu and Trp for 3 days. After successfully growing on double dropout plate, the yeast colonies were picked to plate onto triple dropout minimal medium (SD/-Leu/-Trp/-His). Using positive and negative controls, the possible interactions between *CpbHLH1/2* and *CpCYC-B/CpLCY-B* were defined by their growth status. Y2H experiment protocol was followed. Yeast cells co-transformed PGADT7-*CpbHLH1* + PGBKT7-*CpbHLH2* and PGADT7-*CpbHLH2* + PGBKT7-*CpbHLH1* were grown on triple-dropout and four-dropout minimal medium that basic medium lacks Leu, Trp, His, and Ade.

## Results

### Expression pattern of carotenoid biosynthesis genes during papaya fruit development

**Fig. 1:**
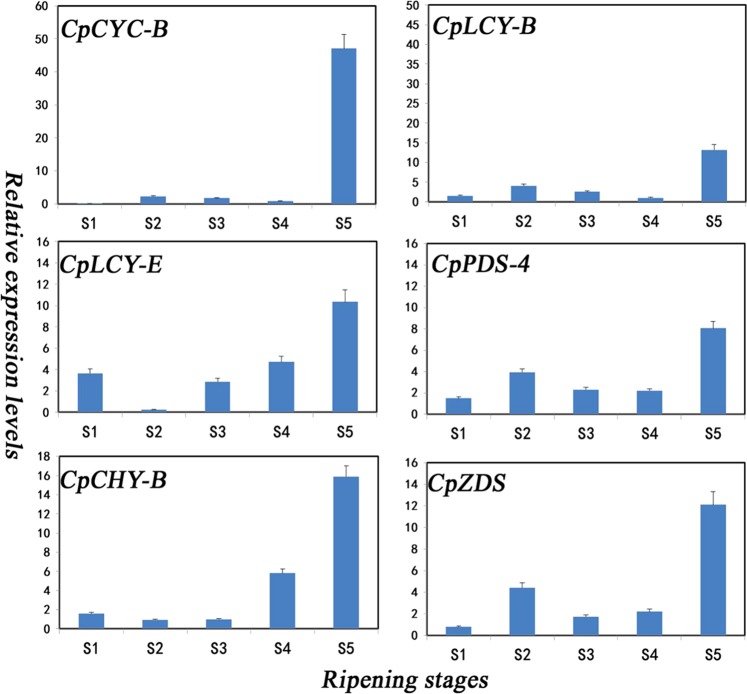
qRT-PCR expression patterns of six carotenoid genes were shown during papaya fruit ripening. S1, 10% ripening; S2, 30% ripening; S3, 50% ripening; S4, 70% ripening; S5, 100% ripening. Value presented as mean + SE of three replicates

The fruit color of papaya is determined by accumulation of different types of carotenoid compositions and content, and the expression level of the carotenoid biosynthesis genes play important roles in papaya flesh color, fruit firmness, and nutritional profile during papaya fruit development. To understand carotenoid biosynthesis genes’ expression pattern during papaya fruit ripening, RNA-Seq analysis was performed to detect carotenoid-related genes’ expression level during five stages of fruit development (Supplement Fig. S2). *CpCYC-B*, *CpCHY-B*, and *CpLCY-B* were significantly upregulated during fruit ripening; *CpZDS*, *CpPDS*, and *CpLCY-E* were also upregulated from stage 3 (50% ripening stage) to stage 5 (100% ripening stage) (Fig. 1).

### Identification and characterization of putative transcription factors involved in *CpCYC-B* and *CpLCY-B* regulation

**Fig. 2:**
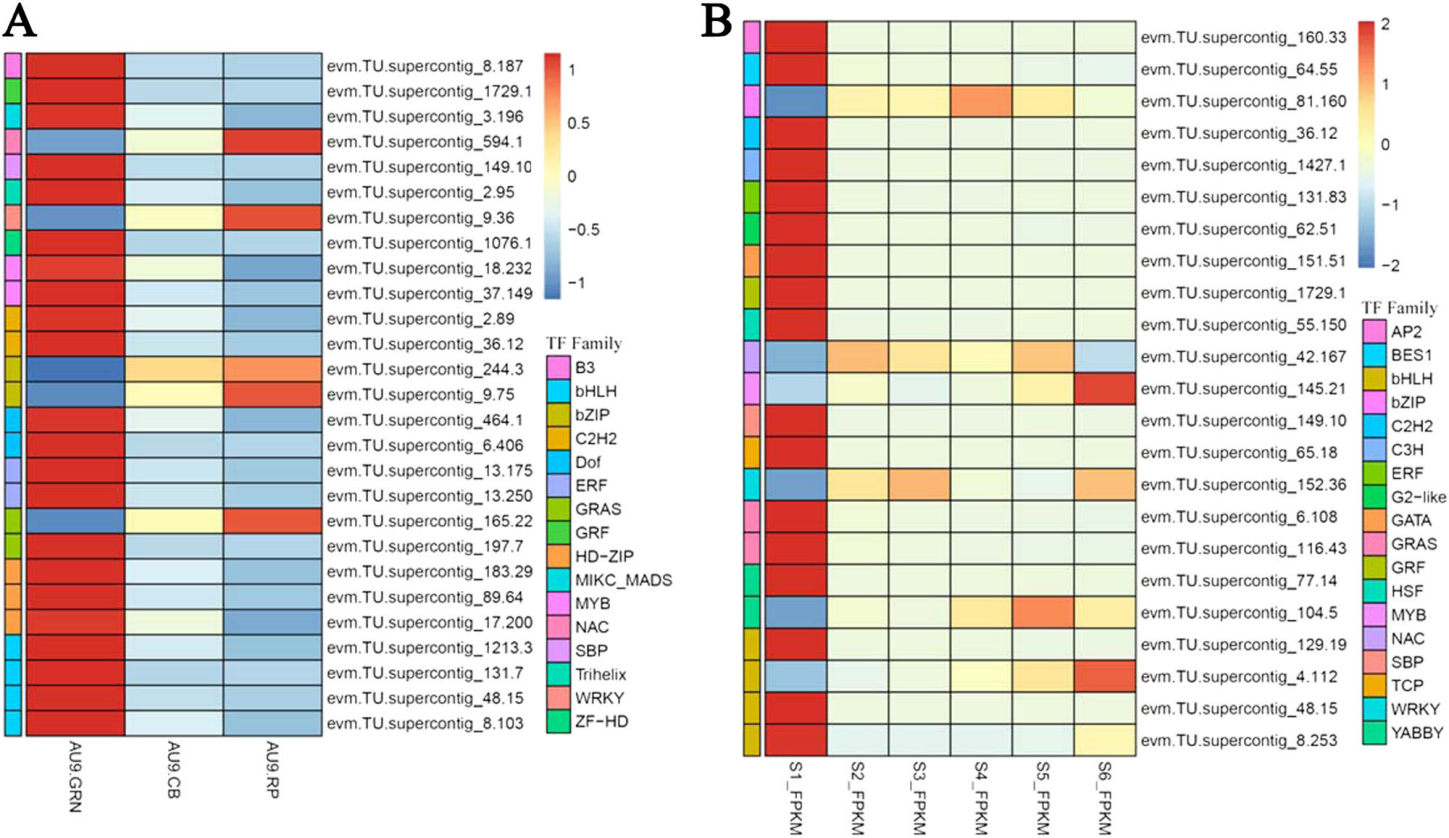
Expression patterns of different expressed transcription factors (DETFs) were shown in two cultivars. **a** Expression pattern of 27 DETFs from green (GRN), color break (CB) to ripeness in AU9 cultivars; **b** Expression pattern of 23 DETFs from stage 1 (S1) to stage 6 (S6) in SunUp cultivars. The cage of red to blue represents quite different expression level. The highly expressed transcription factors were selected for further analysis. Expression value presented as log2FPKM

To further analyze the potential transcription factors regulating carotenoid biosynthesis genes involved in papaya fruit ripening, RNA-Seq analysis was implemented using libraries constructed from papaya varieties SunUp (from S1 to S5) and AU9 (GRN, CB, and RP) fruit at different developmental stages. To calculate reads per kilo bases per million reads (RPKM), significant expressed tags in different samples were identified. The differentially expressed transcription factors in SunUp and AU9 samples were identified based on the following criteria: *P* < 0.01, FDR < 0.01, and fold change >2. Expression profiles of different TFs were classified as bHLH, GRAS, SBP, and bZIP between the first stage and the final stage in SunUp and AU9 cultivars. In total, 23 and 27 different expressed TFs were identified in SunUp and AU9, respectively. Most TFs were downregulated in two cultivars. Of the 23 TFs in Sunup, 17 TFs, including bHLHs, C2H2s, and GRFs, were downregulated, and 5 TFs, including bZIP and B3, were upregulated. Of the 27 TFs in AU9, 21 TFs, including BES1, WRKY, and TCP, were downregulated, and 6 TFs, including bZIP and AP2, were upregulated (Fig. 2). Some of transcription factors were selected for further analysis.

To test whether these transcription factors can bind promoters of *CpCYC-B* and *CpLCY-B* during papaya fruit development, we analyzed the promoter sequences in silico and constructed a series of chimeric genes containing truncated fragments of *CpCYC-B* and *CpLCY-B* promoters and GUS reporter gene. Promoters were analyzed for potential TFs binding sites using the PlantCare database. The potential regulatory elements in upstream −1.5 kb promoters of *CpCYC-B* and *CpLCY-B* have been summarized in Table 1. Most of them are responsive to hormones and light. CGTCA-motif and ERF, bHLH boxs were the consensus *cis*-elements of *CpCYC-B* and *CpLCY-B* promoters. For example, bHLH binding site (-CANNTG-), was found at −113 and −164, respectively, in *CpCYC-B* and *CpLCY-B* promoters. The distribution of different regulatory elements in different region of promoters implied that some special elements played special function at different developmental stages.

**Table 1:** Putative *cis*-regulatory elements related to specific expression present in *CpCYC-B* and *CpLCY-B*

Motif	Sequence	Function	CpCYC-Promoter	CpLCY-Promoter
TCA-element	-GAGAAGAATA-	Responsive to salicylic acid	−506, −536	
CGTCA-motif	-CGTCA-	Responsive to MeJA	−191, −1137, −1249	−426, −451
ERE	-ATTTCAAA-	Ethylene-responsive element	−356, −865	−763, −2985
GARE-motif	-AAACAGA-	Gibberellin-responsive element		−1281
P-box	-CCTTTTG-	Gibberellin-responsive element	−1208	
EIRE	-ATTTCAAA-	Elicitor-responsive element	−627	
ELI-box3	-AAACCAATT-	Elicitor-responsive element	−751	
TGA-element	-AACGAC-	Responsive to auxin		−1108
Bhlh element	-CACCTG/CATTTG-	Light-responsive element	−113	−164
B-zip element	-AGACGT-	Involvement of abscisic acid, light signal	−574	
NAC-element	-CGTG/CATG-	Involvement diverse biological processes, such ripening	−449, −382	

**Fig. 3:**
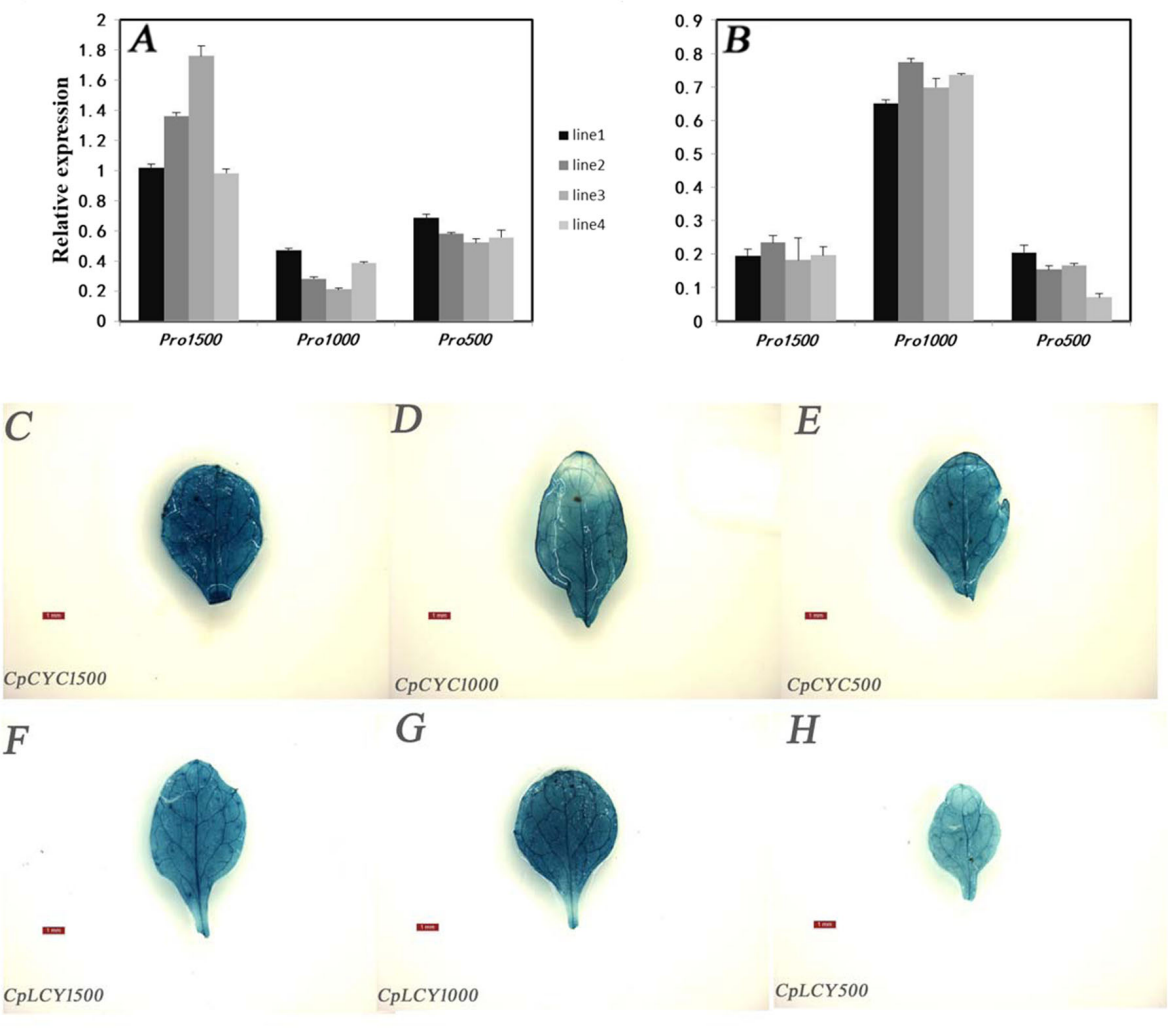
Expression analyses of *CpCYC-B* and *CpLCY-B* promoters were shown in T1 transgenic Arabidopsis. **a** The qRT-PCR expression levels of *GUS* promoted by different promotor promoter fragments of *CpCYC-B* were presented. **b** The qRT-PCR expression levels of *GUS* by different promotor promoter fragments of *CpLCY-B* were presented. The abscissa represents the different promoter lengths; the ordinate represents the qRT-PCR expression level. The expression level was shown as the mean of determinations made with seven to thirteen independent plants. **c**–**h** Histochemical staining of *GUS* activities driven by different promoter fragments of *CpCYC-B* and *CpLCY-B* were obtained from representative transgenic Arabidopsis leaves. **c** *Pro*-1.5k *CpCYC-B*; **d** *Pro*-1.0k *CpCYC-B*; **e** *Pro-*0.5k *CpCYC-B*; **f** *Pro*-1.5k *CpLCY-B*; **g** *Pro*-1.0k *CpLCY-B*; **h** *Pro-*0.5k *CpCYC-B*. Bars represent the standard errors of means. The scale in the figure (**c**–**h**) represents 1 mm

Reporter gene GUS expression was analyzed to check the activity of these *cis*-elements in transgenic Arabidopsis. Higher expression level of GUS gene was detected at −1.5 and −0.5 kb segments of *CpCYC-B* (Fig. 3a). Deletion from −1.5 to −1.0 kb decreased expression compared with the other two deletion promoter segments. The −1.0 kb promoter segment of *CpLCY-B* showed a higher expression level than −1.5 and −0.5 kb promoters. Transgenic plants with pMDC162 alone did not show any GUS expression level (data not shown). These results indicated that −0.5 kb fragment of *CpCYC-B* and *CpLCY-B* promoter, respectively, can activate or inactivate the regulation of GUS gene expression. To further verify GUS gene expression patterns in transgenic lines, Arabidopsis transformed with *CpCYC-B*/*CpLCY-B*-GUS fusion gene were analyzed by histochemical staining (Fig. 3c–h). The GUS activity with −0.5 kb region of *CpCYC-B* and *CpLCY-B* promoter was, respectively, stronger and weaker than another two types of transgenic plants with −1.0 and −1.5 promoters. These results were identical to the relative expression results of different length promoters (Fig. 3a, b), implying that −0.5 kb promoters of *CpCYC-B* and *CpLCY-B* were corresponding to chromoplast- and chloroplast-specific expression patterns, respectively.

We further deleted the −0.5 kb promoters of *CpCYC-B* and *CpLCY-B* into −0.2, −0.3, and −0.4 kb fragments. We discovered that *Pro*-*CpCYC-B::GUS* had a relatively higher expression level with −0.2 kb promoter than other forms of transgenic Arabidopsis. However, *Pro*-*CpLCY-B::GUS* showed similar relative expression levels driven by different promoters fragments (Supplement Fig. S3). In addition, we mutated two elements, -GAAAGAA-(311 bp) and -ATTTCAAA-(ERF-responsive element) in −0.5 kb of *CpLCY-B* and *CpCYC-B* promoters, respectively, and the results indicated that −0.5 kb of *Pro-CpLCY-B::GUS* without -GAAAGAA-element exhibited a relatively higher expression level compared with those containing this element, suggesting -GAAAGAA-element might act as a suppressor in *CpLCY-B* promoter. Without ERF element in −0.5 kb Pro-*CpCYC-B::GUS* T1 transgenic plants, there was no obvious expression difference with other transgenic promoter fragment in Arabidopsis (Supplement Fig. S3A and B). However, when we treated the transgenic Arabidopsis containing −1.0 kb *CpCYC-B* and −1.0 kb *CpLCY-B* promoter with 80 mg/L ethephon, GUS activity appeared to be weaker (Supplement Fig. S3C-H). This result implied that ERF element might act as a negative *cis*-element.

By in silico analysis and experimental validation, we identified that −0.5 kb promoter of *CpCYC-B* and *CpLCY-B* played important regulatory roles and included some fruit-specific elements, e.g., ERF-responsive element, bHLH-box, in addition to the novel negative regulatory element (-GAAAGAA-) in *CpLCY-B* promoter.

**Fig. 4:**
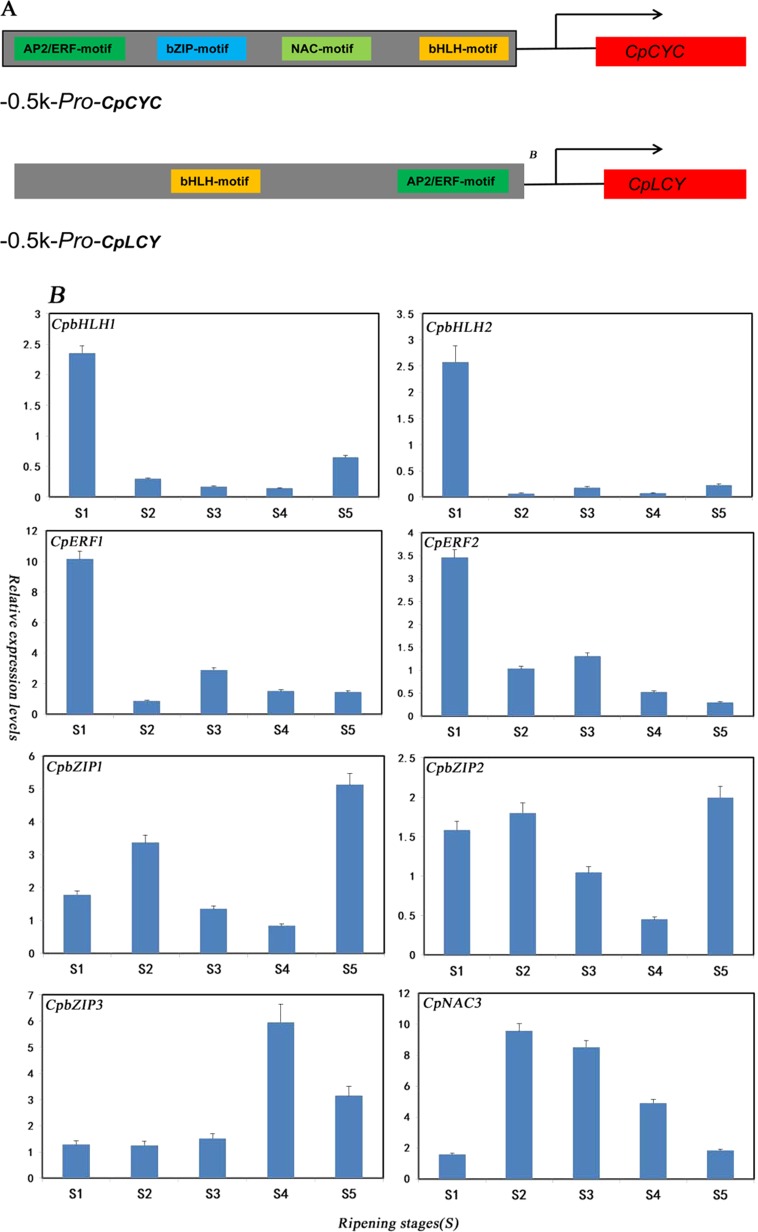
Binding sites and expression pattern of the putative transcription factors were shown during papaya ripening. **a** Different TF familie binding sites existed in *−*0.5k *CpCYC-B* and *CpLCY-B* promoters; **b** qRT-PCR expression patterns of eight transcription factors. The abscissa represents different stages of fruit development and the ordinate represents qRT-PCR expression levels. Value was shown as mean + SE of three replications

Combining the result of the expression of TF family members during different fruit ripening stages and TF family motif in −0.5 kb promoters of *CpCYC-B* and *CpLCY-B*, we obtained four types of transcription factor families as shown in Fig. 4a. According to their distribution in promoters of *CpCYC-B* and *CpLCY-B*, all of their binding sites existed in −0.5 kb *CpCYC-B* promoter, while, bHLH and ERF binding sites existed in −0.5 kb *CpLCY-B* promoter. The characterizations of four TF families were shown in Table 2. Eight TFs belonging to four types of TF families were selected from database (Fig. 2). The eight differentially expressed transcription factors were named as *CpbHLH1* (evm.model.supercontig_131.7), *CpbHLH2* (evm.model.supercontig_8.253), *CpbZIP1* (evm.model.supercontig_81.160), *CpbZIP2* (evm.model.supercontig_9.75), *CpNAC3* (evm.model.Supercontig_594.1), CpERF1 (evm.model.supercontig_131.83), *CpERF2* (evm.model.supercontig_13.250), and *CpbZIP3* (evm.model.supercontig_244.3). The transcription of the eight TFs was validated by RNA-Seq and qRT-PCR. The expression levels of *CpbHLH1* and *CpERF1/2* were decreased from the initial stage. As shown in Fig. 4b, the expression level of *CpNAC3* had been decreased from stage 2; the expression patterns of *CpbZIP1/2/3* were random, and for example, the expression level of *CpbZIP1* first increased, then decreased and finally increased again. For *CpbHLH2*, expression level first increased at color break stage, decreased from stage 2, and then increased significantly from stage 4. These results were further demonstrated by RNA-Seq data (Supplement Fig. S4).

**Table 2:** Characterization of identified transcription factors

TRFs	Motif	Function description of transcript-on factor family
bHLH	CANNTG	Stress response and jasmonic acid signaling, salt stress and cold stress, fruit development, light responsive
bZIP	AGACGT	Involvement of abscisic acid, light signal, environmental stress and seed organ development
AP2/ERF	GCCGCC/GCCG	The response of plants to hormones (ethylene and ABA), pathogens and stresses (low temperature, drought and high salinity) and so on
NAC	CGTG/CATG	Involvement in various biological process such as development, senescence, pigmentation of fruits, stress responses

### *CpbHLH-1/2* binds to *CpCYC-B* and *CpLCY-B* promoters and regulates their expression

**Fig. 5:**
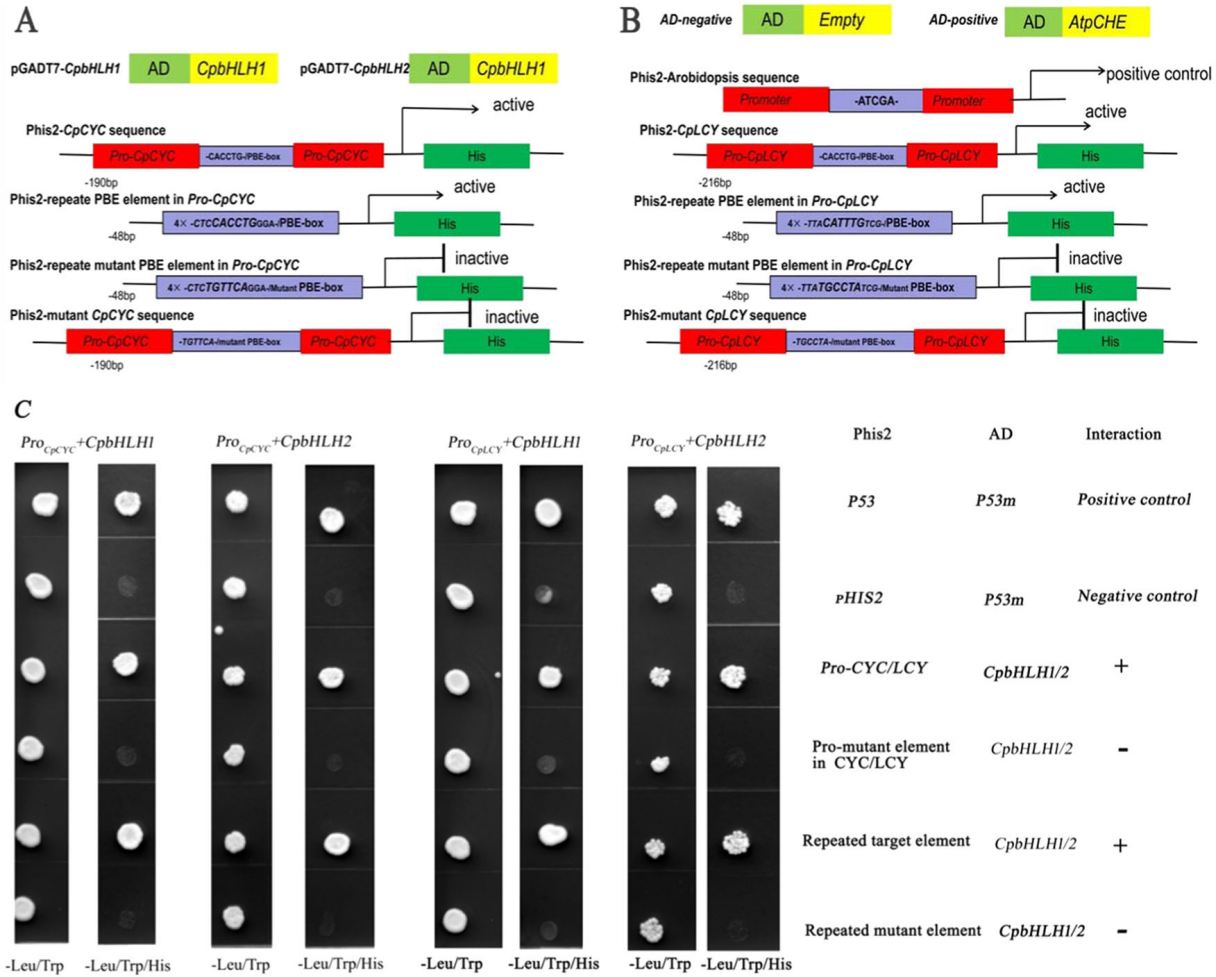
Yeast one-hybrid interactions were tested between *CpbHLH1/2* and *CpCYC-B*/*CpLCY-B*. **a** Schema charts of *CpbHLH1/2* acting with promoters of *CpCYC-B* in different control. **b** Schema chart of *CpbHLH1/2* acting with promoters of *CpLCY-B* in different control. **c** Yeast one-hybrid interactions between *CpbHLH1*/*CpbHLH2* and *CpCYC-B*/*CpLCY-B*. *CpbHLH1/2* interacted with *CpCYC-B*/*CpLCY-B* in Leu-/Trp- and Leu-/Trp-/His-medium with different controls. Interactione were indicated by the ability of yeast cells to grow on a synthetic medium lacking tryptophan, leucine, histidine. Yeast cells transformed with pGADT7-53m + p53HIS were used as a positive control, while those transformed with Phis2 + pGADT7-53m, Phis2-Pro-Mutant element in CYC/LCY + AD-*CpbHLH1/2*, Phis2- repeat mutant element + AD-*CpbHLH1/2* as negative controls. p53m:Pgad53m; p53:p53HIS; Pro-CYC/LCY: selected about 190 bp promoters, including bHLH elements of *CpCYC-B*/*CpLCY-B;* Pro-mutant element in *CYC*/*LCY*: mutant bHLH element in 190 bp promoters of *CpCYC-B*/*CpLCY-B;* Repeated target element: five repetitions of bHLH element sequence; Repeated mutant target element: five repetitions of mutant bHLH element sequence

In view of the expression pattern of eight TFs, it is reasonable to assume that they could regulate the expression of *CpCYC-B* and *CpLCY-B*. To test whether these TFs can bind to the promoters of *CpCYC-B*/*CpLCY-B*, yeast one-hybrid (Y1H) experiment was performed. Yeast cells were co-transformed with the pGADT7-*CpbHLH1* + *CpCYC-B*190bp promoter, pGADT7-*CpbHLH1* + *CpCYC-B*190bp promoter mutant, pGADT7-*CpbHLH1* + target repeated element, pGADT7-*bHLH1* + repeated target mutation element, positive control and negative control would be cultured on basic SD medium lacking Leu and Trp element (Fig. 5c). However, yeast cells co-transformed with the positive control, pGADT7-bHLH1 + CpCYC-B190bp promoter, pGADT7-*bHLH1* + target element repetition, could grow on triple dropout minimal medium. These results were similar to yeast cells co-transformed with pGADT7-*bHLH2* + *CpCYC-B* promoter, pGADT7-*bHLH2* + *CpLCY-B* promoter, pGADT7-*bHLH1* + *CpCYC-B* promoter. These results indicate that *CpbHLH1/2* can bind to CANNTG motifs in *CpCYC-B* and *CpLCY-B* promoters in yeast cells (Fig. 5). However, other six TFs (including *CpbZIP1/2/3*, *CpNAC3*, and *CpERF1/2*) failed to act with promoters of *CpCYC-B*/*CpLCY-B* (Supplement Fig. S5). We also preliminarily proved that *CpbHLH1/2* could bind to CANNTG motif in *CpZDS*, *CpLCY-E*, and *CpCHY-B* promoters though Y1H but fail to bind the promoter of *CpPDS*. Thus, a co-expression pattern existed between *CpbHLH1/2* and *CpZDS*, *CpLCY-E*, *CpCHY-B*, *CpCYC-B*, and *CpLCY-B* (Supplement Fig. S6). Meanwhile, the there was no interaction between *CpbHLH1* and *CpbHLH2* by yeast two-hybrids (Supplement Fig. S7).

**Fig. 6:**
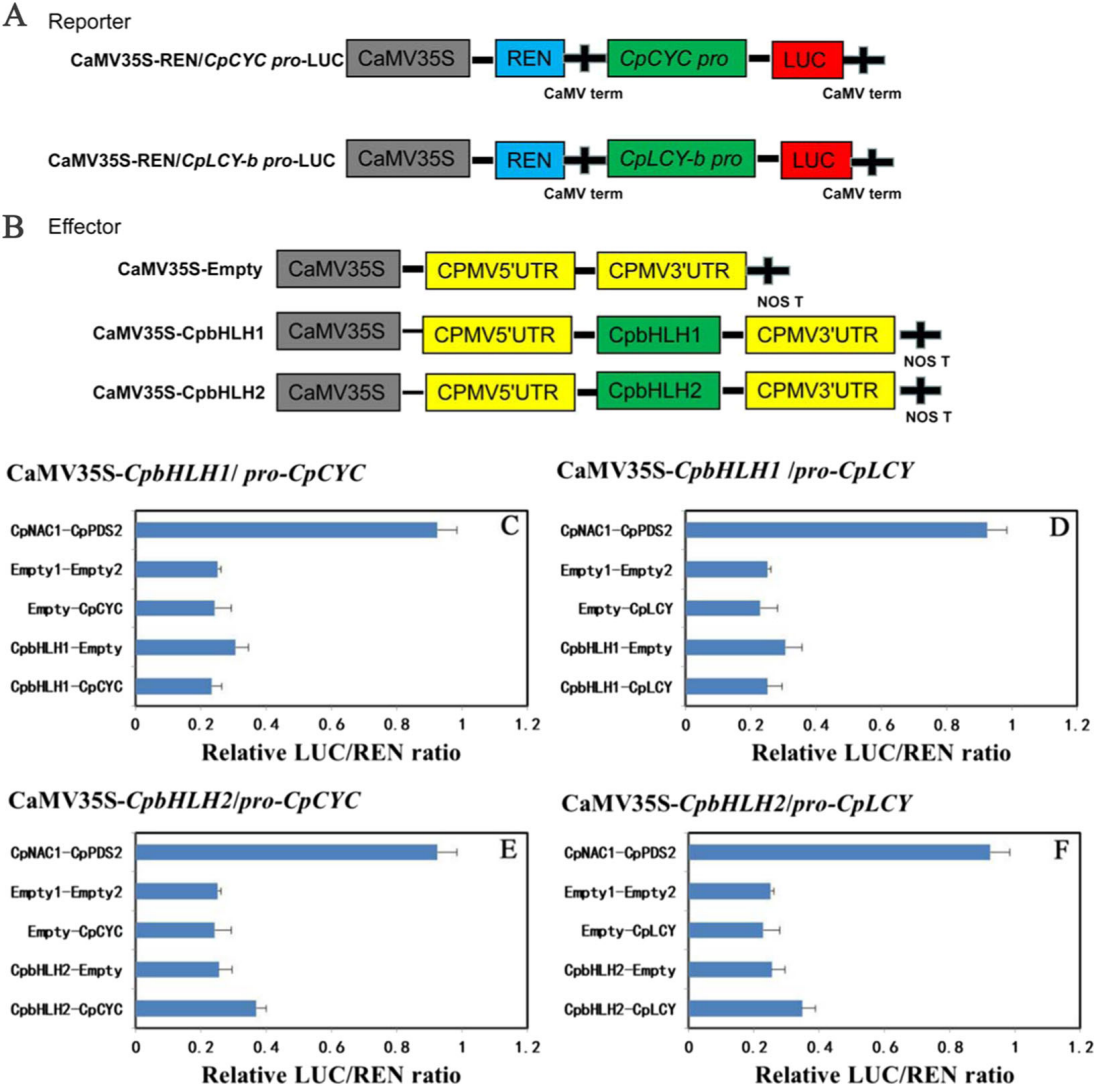
*CpbHLH1* and *CpbHLH2* individually regulated *CpCYC-B* and *CpLCYC-B*. **a**, **b** Vivid figures were shown about reporter and effector constructs according to the dual-luciferase reporter assay. **a** Reporter; **b** Effector; **c**–**f** *CpbHLH1/2* regulated activities of *CpCYC-B* and *CpLCY-B*. **c** *CpbHLH1* repressed *CpCYC-B*; **d** *CpbHLH1* repressed *CpLCY-B*; **e** *CpbHLH2* promoted *CpCYC-B*; **f** *CpbHLH2* promoted *CpLCY-B*; the activation of *CpCYC-B* and *CpLCY-B* by *CpbHLH1/2* were presented by LUC/REN. The ratio of LUC/REN of the empty construct plus promoter vector or transfectors vector was presented. Values showed as mean + SE of eight biological replications

To investigate the effects of *CpbHLH1/2* on *CpCYC-B* and *CpLCY-B* expression, dual-luciferase transient expression assays were performed. The LUC/REN ratio was lower than the positive control and negative control (*CpbHLH1*/Empty) when either the *CpCYC-B* or *CpLCY-B* pro-LUC reporter construct was co-transfected with the CaMV35S-*CpbHLH1* effectors, implying that *CpbHLH1* repressed *CpCYC-B* and *CpLCY-B* expression activities (Fig. 6c, d). In contrast, the LUC/REN ration of *CpbHLH2*-*CpCYC-B*/*CpLCY-B* was higher than the negative control (*CpbHLH1*/Empty) when previous promoters-LUC reporter constructs was co-transfected with the CaMV35S-*CpbHLH2* effectors, indicating that *CpbHLH2* may involve the activated regulation of *CpCYC-B* and *CpLCY-B* (Fig. 6e, f). Besides, LUC/REN ratios of four types of experimental groups were found to be similar to the empty vector controls. We have not performed the transient expression analysis on the other carotenogenesis genes, e.g, *CpZDS*, *CpCHY-B*, and *CpLCY-E*, although bHLH binding sites existed in their promoters (Supplement sequence1). Collectively, these results indicate that *CpbHLH1* and *CpbHLH2* could individually repress and promote *CpCYC-B* and *CpLCY-B* genes during papaya fruit ripening.

### *CpbHLH1/2* involved in expression regulation of *CpCYC-B* and *CpLCY-B* responded to light

**Fig. 7:**
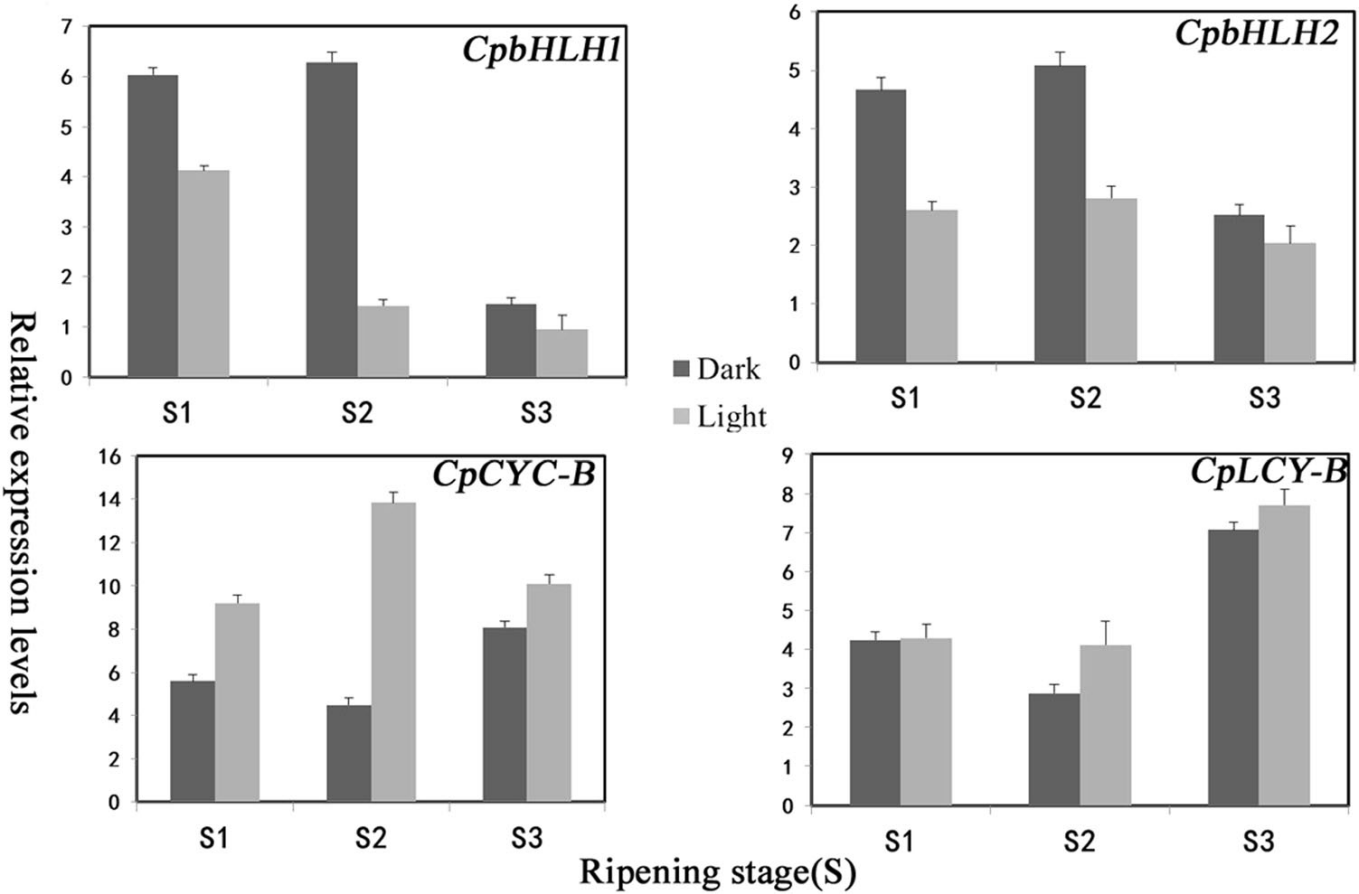
Under the treatment of 2-day dark and white light, the qRT-PCR expressions of four genes were examined from stages 1 to 3. The abscissa represents different developmental stages of papaya fruits, and the ordinate means qRT-PCR expression level. Stage 1: green stage; stage 2: color break; stage 3: ripe

To investigate if *CpbHLH1/2* could regulate gene expression on carotenoid biosynthesis pathway (including *CpCYC-B* and *CpLCY-B*) in response to light, we collected SunUp fruit from S1 to S3 stage (green, color break, and ripe). Under 28 °C, papaya fruits of each stage were divided into two parts, one part was exposed to strong white light for 2 days, another part was kept in dark. After 2 days of treatment, expression levels of *CpbHLH1/2*, *CpCYC-B*, and *CpLCY-B* were examined by qRT-PCR. In Fig. 7, the expression levels of *CpCYC-B* and *CpLCY-B* in light were higher than in dark. In contrast, the expression levels of *CpbHLH1/2* were much lower in light than in dark. Especially, at stages S1 and S2 when the carotenoids have not been accumulated, the expression levels of *CpCYC-B* and *CpbHLH2* were twice in light and in dark, respectively. At ripe stage S3, there was no obvious expression difference for *CpbHLH1/2*, *CpCYC-B*, and *CpLCY-B*. In conclusion, *CpbHLH1/2* may be among the factors regulating *CpCYC-B* and *CpLCY-B* in response to light.

## Discussion

Carotenoids are very important pigments for plant growth and development, contributing to fruit flesh color and nutritional properties, and protecting the photosynthetic apparatus. In fruit, carotenoid accumulation could be promoted by light, but also could be harmed by excess light. The way their production is regulated by light is common^21,33–35^. Lycopene conversion into carotenoids in ripening papaya flesh was regulated by *CpbHLH1/2* based on a molecular mechanism that is similar in Arabidopsis leaves or other plants in response to light^24^. In red grapefruit, lycopene content in shaded fruits is 49-fold than fruit exposing to light, implying shading- promoted carotenoid accumulation^36^.

During papaya fruits ripening, transcription factors play a significant role in carotenoid biosynthesis and enhance carotenoid accumulation (Fig. 1). In order to identify TFs related to papaya carotenoids biosynthesis, we analyzed RNA-Seq data of different papaya fruit in SunUp and AU9, especially the period during color break stage when the content of carotenoids changes sharply. More than 40 TF genes were identified that are positively or negatively regulated during papaya fruit development (Fig. 2). These TF genes belong to NAC, bHLH, ERF, bZIP, Dof, MYB, SBP, and C2H2. To find the possible TF families binding to the *CpCYC-B* and *CpLCY-B* promoters, these promoters were identified and isolated. By analysis of promoter fragments, −0.5 kb promoter sequences of *CpCYC-B* and *CpLCY-B* were considered as the effective region in response to fruit ripening (Fig. 3). Binding sites of NAC, bHLH, bZIP, and ERF were found in −0.5 kb promoter of *CpCYC-B*, while binding sites of bHLH and bZIP were found in −0.5 kb in *CpLCY-B* (Table 2). In previous studies, all of these TF families are involved in light response. In Arabidopsis, responding to light, *bZIP16* both negatively inhibit the cell elongation and positively promote seed germination^37^. In tomato transgenic plants containing the main ORFs of *SlbZIP1* and *SlbZIP2*, the sugar content (sucrose/glucose/fructose) was 1.5-fold higher than non-transgenic fruits because *SlbZIP* *trans*-activates the asparagine synthase and proline dehydrogenase genes^38^. In Citrullus colocynthis, higher transcripts of *CcNAC1* and *CcNAC2* following red light imply that NAC genes might function in light signaling pathway^39^. Besides, *CpNAC1* was found to play a positive regulatory role in carotenoid accumulation through boosting *CpPDS2/4* transcription by direct binding to their promoters^34^. When transferring Arabidopsis thaliana triose phosphate/phosphate translocator (*tpt*) mutants from low light to high light, transcripts expression level of four AP2/ERF genes, *ERF6*, *RRTF1*, *ERF104*, and *ERF105* will absolutely and quickly decreased within 10 min, implying that AP2/ERF-TFs were responsive to strong light^40^. Through Y1H experiments, *CpbHLH1/2* could bind to *CpCYC-B* and *CpLCY-B* promoters (Fig. 5). This suggested that *CpbHLH1/2* are required to regulate the expression of *CpCYC-B* and *CpLCY-B*.

In papaya, the transcriptional regulation mechanism of carotenogenesis mediated by bHLHs is unclear. In current research, three models of transcriptional regulation by bHLHs were proposed. First, in dark or etiolated seedlings, responding to shade or night, PIFs mainly act as constitutive transcriptional activators. Under diurnal conditions, reversely, light will weaken or remove the activation of PIFs. A considerable portion of PIF-associated genes are upregulated in *pif* mutants, particularly during etiolating process, implying that *PIFs* repress the expression of those genes^41,42^. Second, during de-etiolation, *PIFs* also repress the constitutive transcription of a comparatively small subset of light-induced genes, which indicated that PIFs might have two function depending on the promoter sequences and environment cues. For example, *PIF1* repressed the expression of *PSY* and *CBF2* in dark or diurnal conditions^24,43,44^, and *PIF1* and *PIF3* also could inactivate the genes associated with chlorophyll biosynthesis and photosynthesis during de-etiolation^45,46^. In the third model, *PIFs* constitutively co-activate momently light-induced genes such as *ELIP2* during de-etiolation, suggesting other undiscovered transcriptional co-activator may participate in this process. In the dark, PIFs do not repress early light-induced genes like *ELIP1* and *ELIP2*, but PIFs are required for their rapid light induction^47,48^. Acting as a co-activator, *PIFs* respond to light positively and lead to decrease transient expression of *ELIP2*. This model may also conduct for a broader spectrum of genes, perhaps some light-responsive genes, *SIGE* and *ELIP* genes, which are not regulated by PIFs in dark or light. In our results, the regulatory mechanism of *CpbHLH-1/2* was similar to previous models. From results in Fig. 7, we peopose two bHLH mediated-models: (1) light activate Pr phytochrome into Pfr, resulting in the degradation of *CpbHLH1*, thus there was no active *CpbHLH1* to inhibit the expression of *CpCYC-B* and *CpLCY-B* during strong white light as in the first model; (2) *CpbHLH2* might co-activate *CpCYC-B* and *CpLCY-B* expression with other activators. Protein–protein interaction assays suggested that *CpbHLH1* could not physically interact with *CpbHLH2*, that they could not co-operatively regulate *CpCYC-B* and *CpLCY-B* (Supplement Fig. S7).

In previous studies, *PSY* was reported to be the only one gene that was controlled by PIF transcription factors involving light-mediated regulation in carotenoid pathway, because it is considered as the main-rate limiting enzyme of carotenoid pathway^23^. In current study, bHLH family members, *CpbHLH1* and *CpbHLH2* bind promoter regions of *CpCYC-B* and *CpLCY-B* and regulate their expression during papaya fruit development. Correlating with the decreased expression of *CpbHLH1/2*, expression level of the *CpCYC-B* and *CpLCY-B* was elevated during fruit ripening. We analyzed transcriptional regulation network and identified some potential family members regulating carotenoid biosynthesis genes in response to light or other factors during papaya fruit development in a genome scale. Our results first reported bHLH family members involved in the regulated network of carotenoid biosynthesis-related genes in papaya, which revealed two novel TFs of bHLHs and their regulation mechanism related to carotenoid biosynthesis genes in response to light. These findings contribute to uncover regulatory cascades, which could be directly or indirectly regulate fruit ripening in papaya responding to light.

## Supplementary information

Supplementary figures

Supplementary Tables

## References

[1] Klee HJ, Giovannoni JJ (2011). Genetics and control of tomato fruit ripening and quality attributes. Annu. Rev. Genet..

[2] Martel C (2015). Tomato whole genome transcriptional response to Tetranychus urticae identifies divergence of spider mite-induced responses between tomato and Arabidopsis. Mol. Plant-Microbe Inter..

[3] Dumas Y, Dadomo M, Di Lucca G, Grolier P (2003). Effects of environmental factors and agricultural techniques on antioxidant content of tomatoes. J. Sci. Food Agric..

[4] Park H, Kreunen SS, Cuttriss AJ, Dellapenna D, Pogson BJ (2002). Identification of the carotenoid isomerase provides insight into carotenoid. Biosynth., Prolamellar Body Form., Photo..

[5] Oa I, Li F, Murillo C, Wurtzel ET (2007). Maize Y9 encodes a product essential for 15-*cis*-ζ-carotene isomerization. Plant Physiol..

[6] Chen Y., Li F., Wurtzel E. T. (2010). Isolation and Characterization of the Z-ISO Gene Encoding a Missing Component of Carotenoid Biosynthesis in Plants. PLANT PHYSIOLOGY.

[7] Nisar N, Li L, Lu S, Khin NC, Pogson BJ (2015). Carotenoid metabolism in plants. Mol. Plant.

[8] Yuan H, Zhang J, Nageswaran D, Li L (2015). Carotenoid metabolism and regulation in horticultural crops. Hortic. Res.

[9] Manning K (2006). A naturally occurring epigenetic mutation in a gene encoding an SBP-box transcription factor inhibits tomato fruit ripening. Nat. Genet..

[10] Martel C, Vrebalov J, Tafelmeyer P, Giovannoni JJ (2011). The tomato MADS-box transcription factor RIPENING INHIBITOR interacts with promoters involved in numerous ripening processes in a COLORLESS NONRIPENING-dependent manner. Plant Physiol..

[11] Chung MY (2010). A tomato (*Solanum lycopersicum*) *APETALA2/ERF* gene, SlAP2a, is a negative regulator of fruit ripening. Plant J..

[12] Karlova R (2011). Transcriptome and metabolite profiling show that APETALA2a is a major regulator of tomato fruit ripening. Plant Cell.

[13] Lee JM (2012). Combined transcriptome, genetic diversity and metabolite profiling in tomato fruit reveals that the ethylene response factor SlERF6 plays an important role in ripening and carotenoid accumulation. Plant J..

[14] Welsch R, Maass D, Voegel T, DellaPenna D, Beyer P (2007). Transcription factor RAP2.2 and its interacting partner SINAT2: stable elements in the carotenogenesis of Arabidopsis leaves. Plant Physiol..

[15] Zhu M (2014). A new tomato NAC (NAM ATAF1/2/CUC2) transcription factor, SlNAC4, functions as a positive regulator of fruit ripening and carotenoid accumulation. Plant Cell Physiol..

[16] Lu Swen (2018). The citrus transcription factor CsMADS6 modulates carotenoid metabolism by directly regulating carotenogenic genes. Plant Physiol..

[17] Sagawa JM (2016). An R2R3-MYB transcription factor regulates carotenoid pigmentation in Mimulus lewisii flowers. New Phytol..

[18] Vrebalov J (2009). Fleshy fruit expansion and ripening are regulated by the tomato SHATTERPROOF gene TAGL1. Plant Cell.

[19] Zhou T (2012). Virus-induced gene complementation reveals a transcription factor network in modulation of tomato fruit ripening. Sci. Rep..

[20] Leivar P, Monte E, Cohn MM, Quail PH (2012). Phytochrome signaling in green Arabidopsis seedlings: Impact assessment of a mutually negative phyB-PIF feedback loop. Mol. Plant.

[21] Azari R (2010). Overexpression of UV-DAMAGED DNA BINDING PROTEIN 1 links plant development and phytonutrient accumulation in high pigment-1 tomato. J. Exp. Bot..

[22] Kilambi HV, Kumar R, Sharma R, Sreelakshmi Y (2013). Chromoplast-specific carotenoid-associated protein appears to be important for enhanced accumulation of carotenoids in hp1 tomato fruits. Plant Physiol..

[23] Llorente B (2016). Tomato fruit carotenoid biosynthesis is adjusted to actual ripening progression by a light-dependent mechanism. Plant J..

[24] Toledo-Ortiz G, Huq E, Rodriguez-Concepcion M (2010). Direct regulation of phytoene synthase gene expression and carotenoid biosynthesis by phytochrome-interacting factors. Proc. Natl Acad. Sci. USA.

[25] Hornitschek P, Lorrain S, Zoete V, Michielin O, Fankhauser C (2009). Inhibition of the shade avoidance response by formation of non-DNA binding bHLH heterodimers. EMBO J..

[26] Hao Y, Oh E, Choi G, Liang Z, Wang ZY (2012). Interactions between HLH and bHLH factors modulate light-regulated plant development. Mol. Plant.

[27] Hornitschek P (2012). Phytochrome interacting factors 4 and 5 control seedling growth in changing light conditions by directly controlling auxin signaling. Plant J..

[28] Shi H (2013). HFR1 sequesters PIF1 to govern the transcriptional network underlying light-initiated seed germination in Arabidopsis. Plant Cell.

[29] Blas AL (2010). Cloning of the papaya chromoplast-specific lycopene -cyclase, CpCYC-b, controlling fruit flesh color reveals conserved microsynteny and a recombination hot spot. Plant Physiol..

[30] Livak KJ, Schmittgen TD (2001). Analysis of relative gene expression data using real-time quantitative PCR and the 2-ΔΔCT method. Methods.

[31] Zhu X (2012). Evaluation of new reference genes in papaya for accurate transcript normalization under different experimental conditions. PLoS ONE.

[32] Hellens RP (2005). Transient expression vectors for functional genomics, quantification of promoter activity and RNA silencing in plants. Plant Methods.

[33] Fraser PD, Bramley PM (2004). The biosynthesis and nutritional uses of carotenoids. Prog. Lipid Res..

[34] Fu CC (2016). The papaya transcription factor CpNAC1 modulates carotenoid biosynthesis through activating phytoene desaturase genes CpPDS2/4 during fruit ripening. J. Agric. Food Chem..

[35] Ruiz-Sola MÁ, Rodríguez-Concepción M (2012). Carotenoid biosynthesis in Arabidopsis: a colorful pathway. Arab. B..

[36] Lado J (2015). Fruit shading enhances peel color, carotenes accumulation and chromoplast differentiation in red grapefruit. Physiol. Plant..

[37] Hsieh W-P, Hsieh H-L, Wu S-H (2012). *Arabidopsis* bZIP16 transcription factor integrates light and hormone signaling pathways to regulate early seedling development. Plant Cell.

[38] Sagor GHM (2016). A novel strategy to produce sweeter tomato fruits with high sugar contents by fruit-specific expression of a single bZIP transcription factor gene. Plant Biotechnol. J..

[39] Wang Z, Rashotte AM, Dane F (2014). Citrullus colocynthis NAC transcription factors CcNAC1 and CcNAC2 are involved in light and auxin signaling. Plant Cell Rep..

[40] Vogel MO (2014). Fast retrograde signaling in response to high light involves metabolite export, MITOGEN-ACTIVATED PROTEIN KINASE6, and AP2/ERF transcription factors in Arabidopsis. Plant Cell.

[41] Oh E (2009). Genome-wide analysis of genes targeted by PHYTOCHROME INTERACTING FACTOR 3-LIKE5 during seed germination in Arabidopsis. Plant Cell Online.

[42] Zhang Y (2013). A quartet of PIF bHLH factors provides a transcriptionally centered signaling hub that regulates seedling morphogenesis through differential expression-patterning of shared target genes in Arabidopsis. PLoS Genet..

[43] Kidokoro S (2009). The Phytochrome-interacting factor PIF7 negatively regulates DREB1 expression under circadian control in Arabidopsis. Plant Physiol..

[44] Lee C-M, Thomashow MF (2012). Photoperiodic regulation of the C-repeat binding factor (CBF) cold acclimation pathway and freezing tolerance in Arabidopsis thaliana. Proc. Natl Acad. Sci. USA.

[45] Chen D (2013). Antagonistic basic helix-loop-helix/bZIP transcription factors form transcriptional modules that integrate light and reactive oxygen species signaling in *Arabidopsis*. Plant Cell.

[46] Liu T, Carlsson J, Takeuchi T, Newton L, Farré EM (2013). Direct regulation of abiotic responses by the Arabidopsis circadian clock component PRR7. Plant J..

[47] Al-Sady B, Kikis EA, Monte E, Quail PH (2008). Mechanistic duality of transcription factor function in phytochrome signaling. Proc. Natl Acad. Sci. USA.

[48] Leivar P (2009). Definition of early transcriptional circuitry involved in light-induced reversal of PIF-imposed repression of photomorphogenesis in young Arabidopsis seedlings. Plant Cell.

