# Supplementary material for: Papaya CpbHLH1/2 regulate carotenoid biosynthesis-related genes during papaya fruit ripening
Source: Hortic Res. 2019 Jun 22;6:80. doi: 10.1038/s41438-019-0162-2 (PMC6588581; doi:10.1038/s41438-019-0162-2)
Supplement: Supplementary file 1 — Supplementary figures [file 41438_2019_162_MOESM1_ESM.docx]

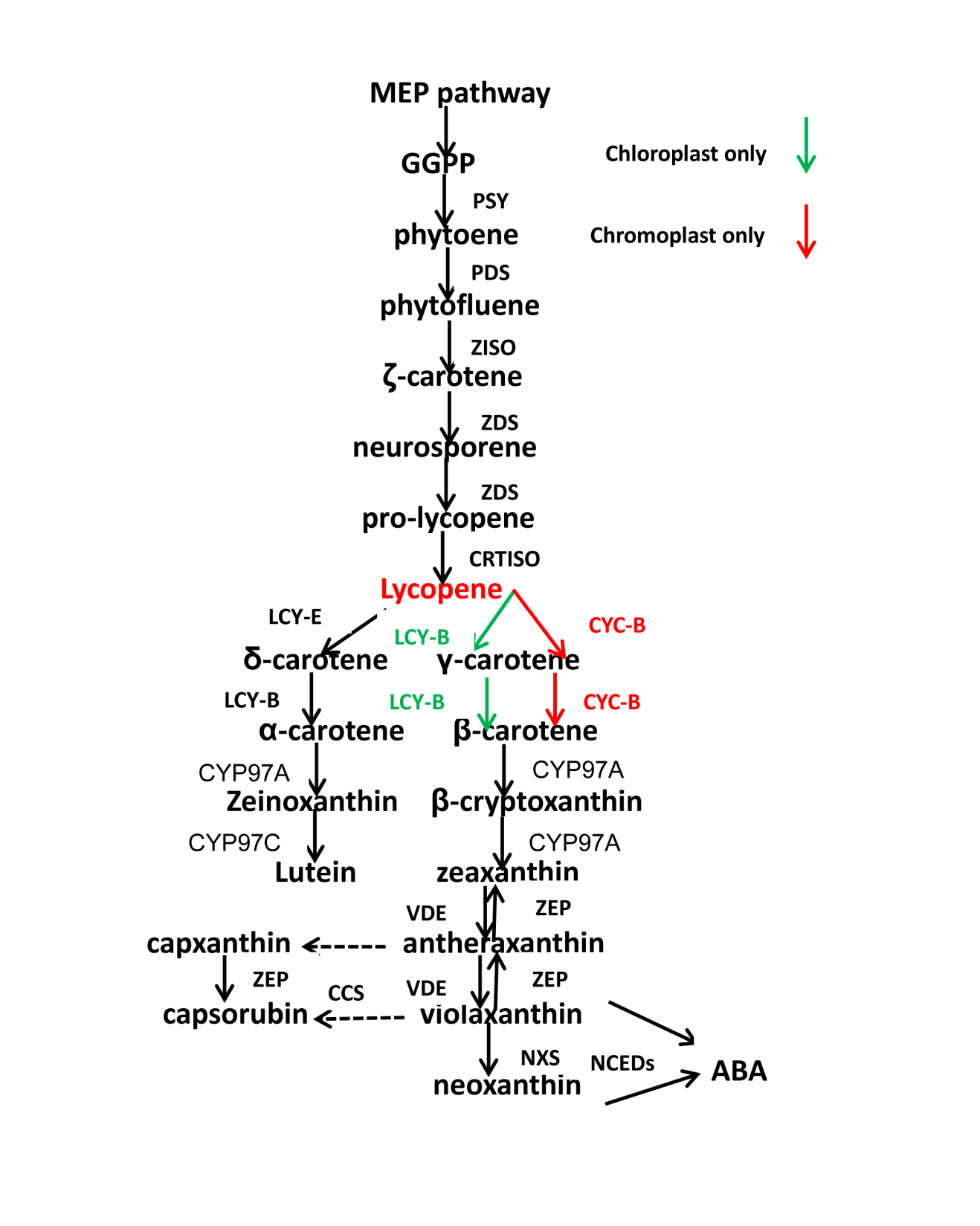


Fig.S1. Carotenoid biosynthesis pathway in papaya fruit is adopted from Skelton et al (2006) and Nisar et al (2015). PSY, phytoene synthesis; PDS, phytoene desaturase; ZISO, ζ-carotene isomerase; ZDS, ζ-carotene desaturase; CRTISO, carotene isomerase; LCY-B, chloroplast-specific lycopene β-cyclase; LCY-E, lycopene ε-cyclase; CYC-B, chromoplast-specific lycopene β-cyclase; CYP97C, e-carotene hydroxylase; CYP97A, 8-carotene hydroxylase; NXS, neoxanthin synthesis; CCS, capsanthin-capsorubin synthase; ZEP, zeaxanthin epoxidase; VDE, violaxanthin de-epoxidase; NCEDs, 9-cis-epoxycarotenoid dioxygenase. Carotenoids are synthesized in the plasids from two molecules of geranylgeranyl diphosphate (GGPP), which are condensed into phytoene by PSY. Phytoene is then converted into lycopene via the intermediate ζ-carotene product involving the action of PDS and ZDS. In red-fleshed papaya, the red lycopene is the major accumulating carotenoid compound. Lycopene is the precursor for two biochemical pathways, leading to lutein via α-carotene and/or abscisic acid via β-carotene and a series of xanthophylls.


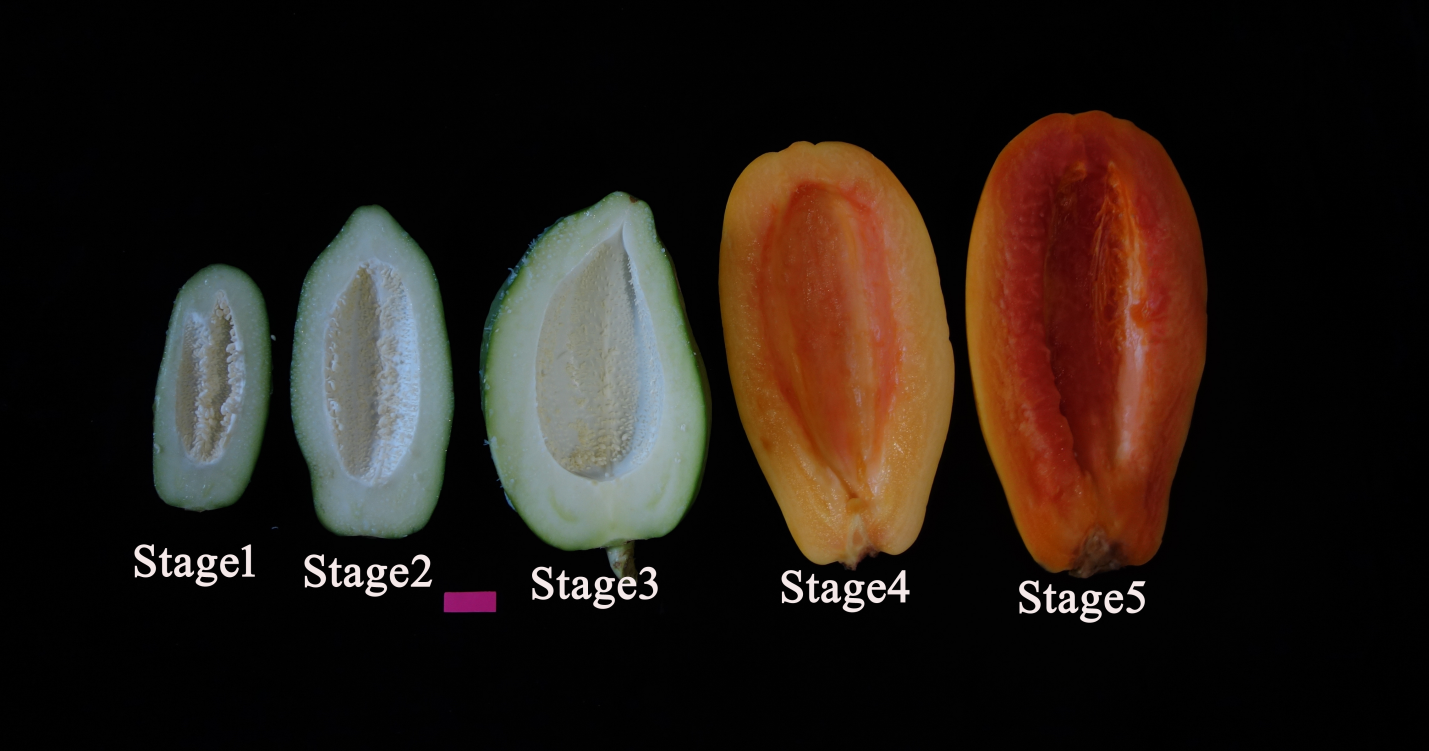


Fig.S2. Different stages of papaya fruit were shown (from S1 to S5). S1, 10% ripening; S2, 30% ripening; S3, 50%ripening; S4, 70%ripening; S5, 100% ripening. The scale represents a unit length of 4 centimeters.


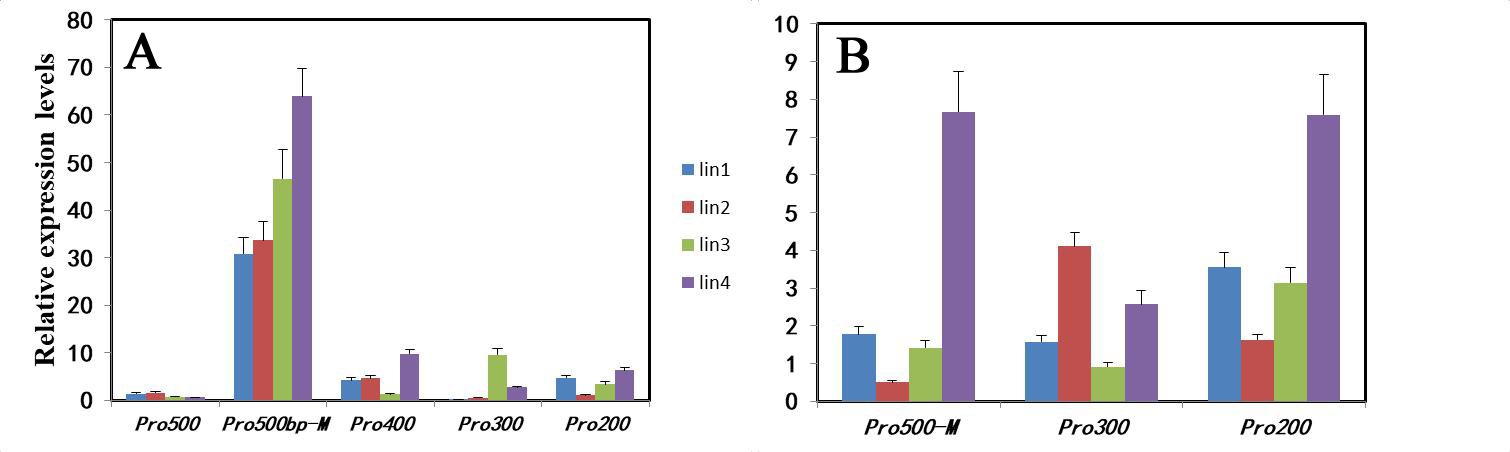


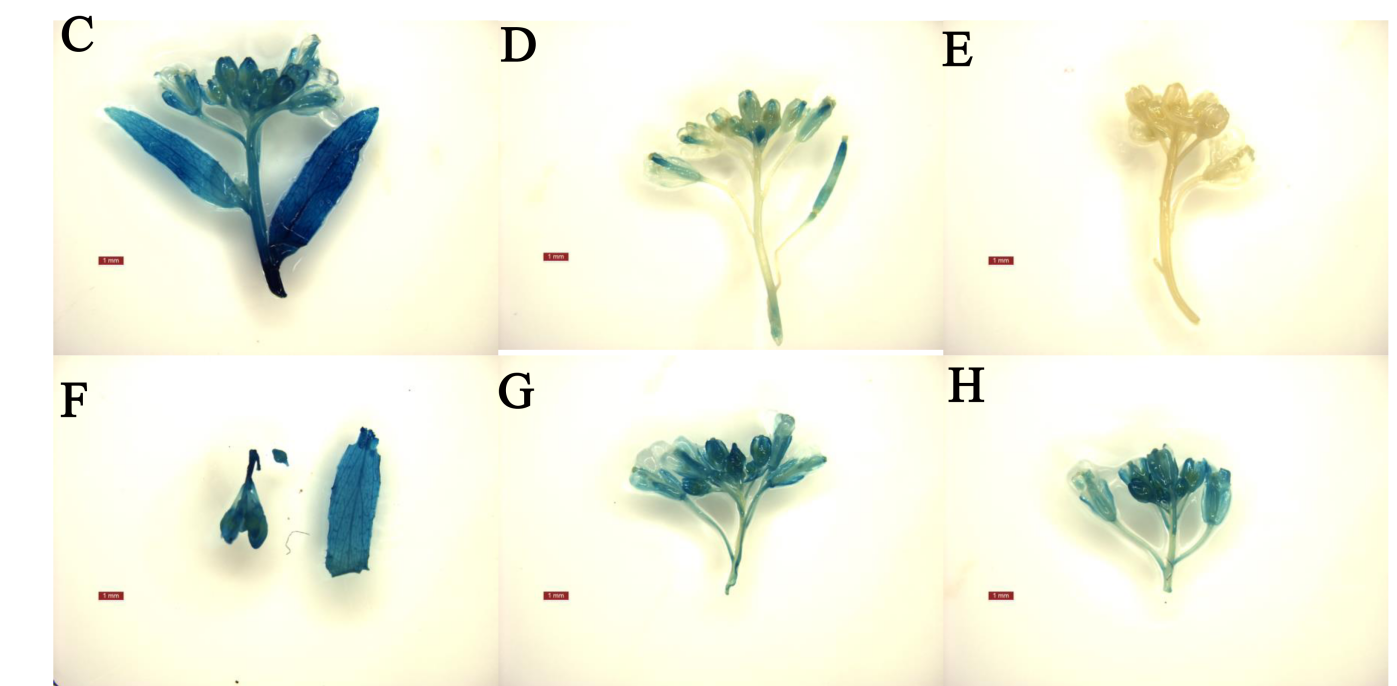


Fig.S3. The expression pattern of *CpCYC-B*/*CpLCY–B* promoters and ethephon treatment were shown. (A-B) qRT-PCR expression was driven by Pro-*CpCYC-B*/*CpLCY–B* from -0.2K to -0.5K, (A) Pro-*CpCYC-B*, (B) Pro-*CpLCY–B.* The abscissa represents the different promoter lengths; the ordinate represents the qRT-PCR expression levels. *Pro-500M* means an element was mutated in 500bp promoter. (C-H) Histochemical staining analysis of Pro-0.1Kb *CpCYC-B* and *CpLCY–B* transgenic Arabidopsis was represented through 80mg/L ethephon treatment. (C) Pro-0.1k*CpCYC-B*-0h; (D) Pro-0.1k*CpCYC-B*-6h; (E) Pro-0.1k*CpCYC-B*-24h; (F) Pro-0.1k*CpLCY-B*-0h; (G) Pro-0.1k*CpLCY-B*-6h; (H) Pro-0.1k*CpLCY-B*-24h. Bars represent the standard error of mean. The scale in the figure C-H represents 1 mm.


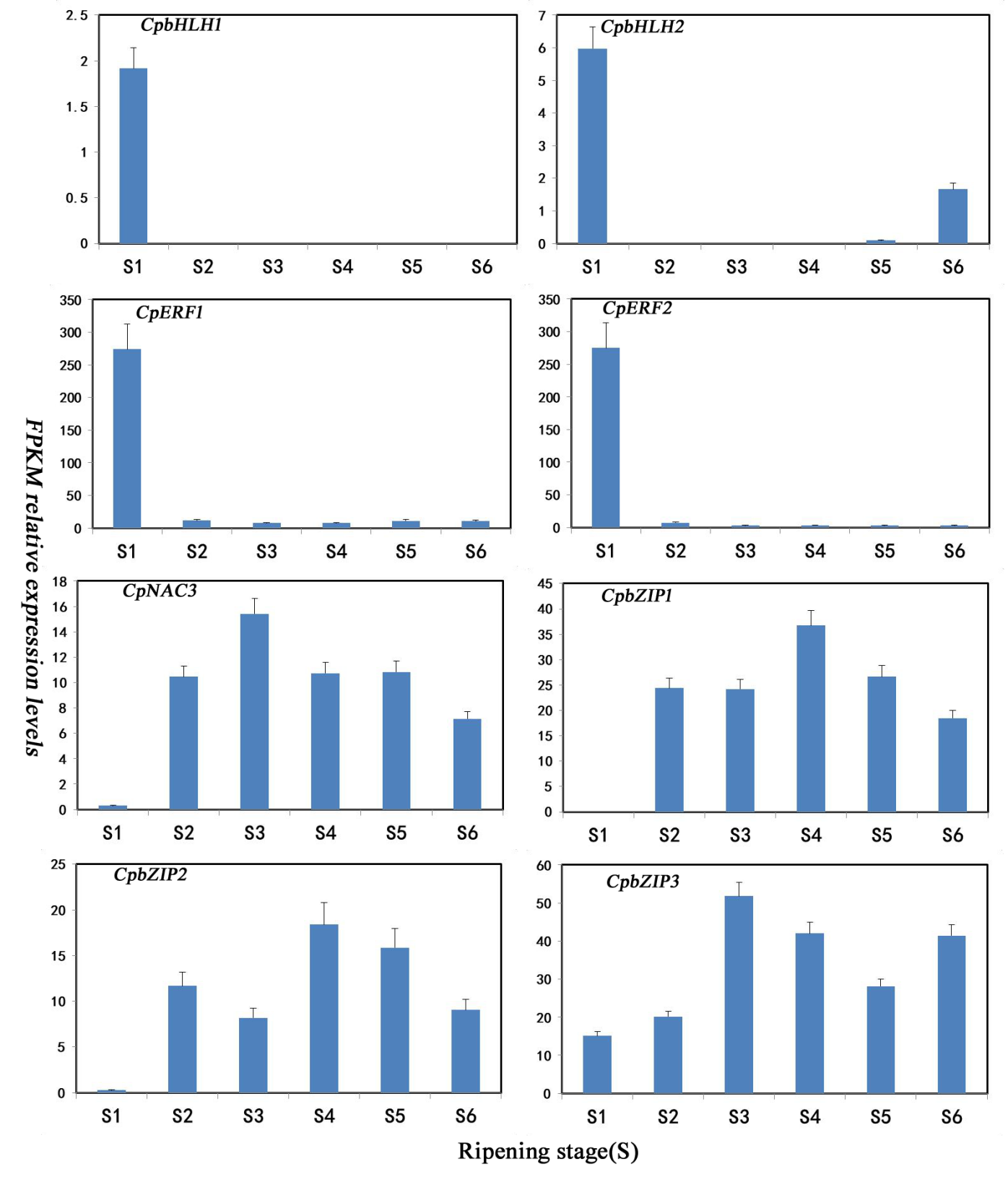


Fig.S4. The FPKM expression patterns of potential functional transcription factors were shown. The abscissa represents different stages of fruit development and the ordinate represents FPKM expression levels. Value was shown as mean +SE of three replications.


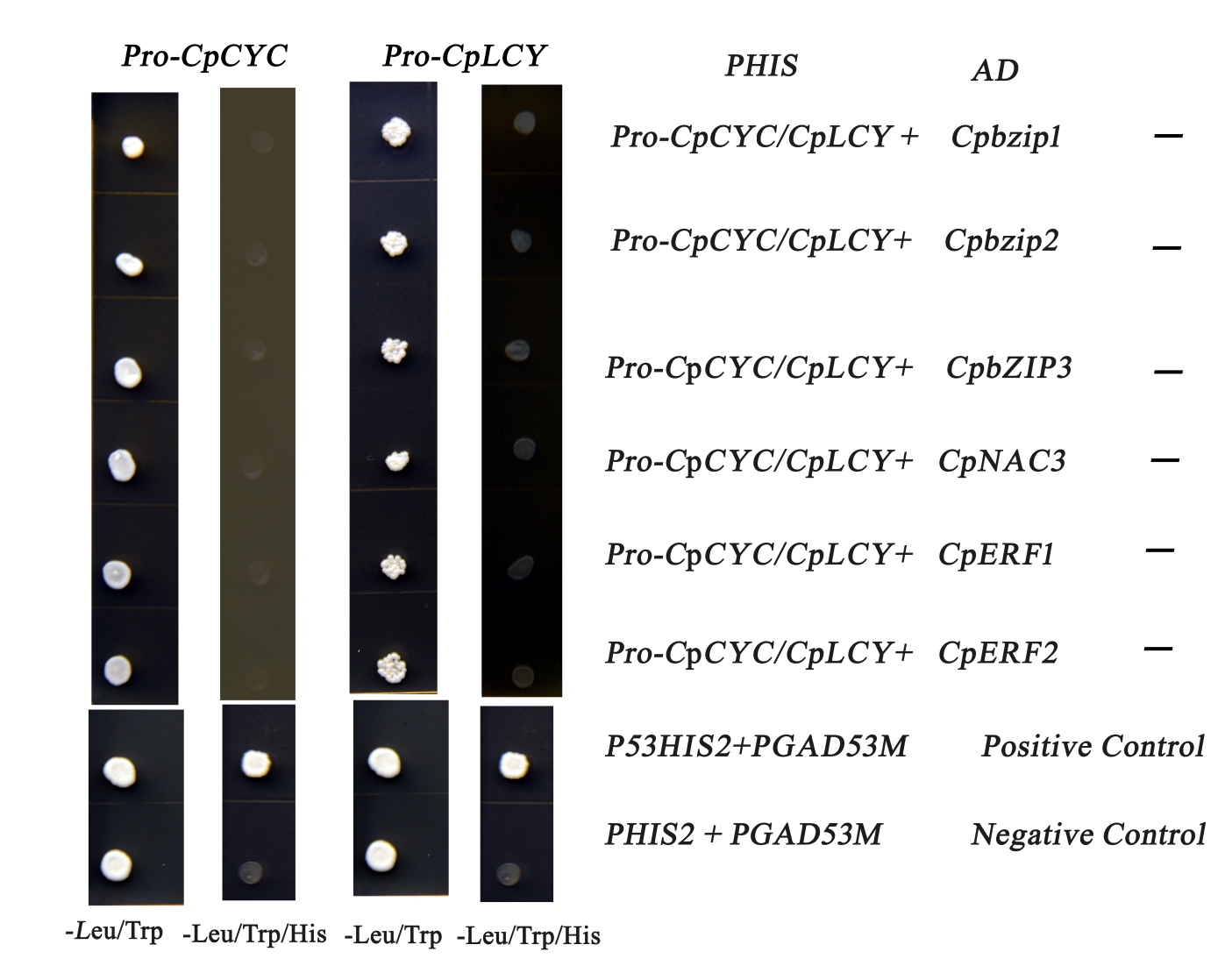


Fig.S5.The yeast one-hybrid interactions were not detected between *CpbZIP1/2/3*, *CpNAC3*, *CpERF1/2* and *CpCYC-B*/*CpLCY-B*. All vectors co-operatively transformed into yeast could grow on SD medium without Leu/Trp, but they couldn’t interact on SD without Leu/Trp/His. Interaction was indicated by the ability of yeast cells to grow on a synthetic medium lacking tryptophan, leucine, histidine. Yeast cells transformed with PGADT7-53M +P53HIS2 were used as positive controls, while those transformed with Phis+pGAD53M as negative controls. *Pro-CpCYC*/*CpLCY*: selected promoters including elements of bZIP, NAC and ERF in *CpCYC-B*/*CpLCY-B*; *CpbZIP1/2/3*: PGADT7- *CpbZIP1/2/3*; *CpNAC3*: PGADT7- *CpNAC3*; *CpERF1/2*: PGADT7- *CpERF1/2;* -*:* inactive.


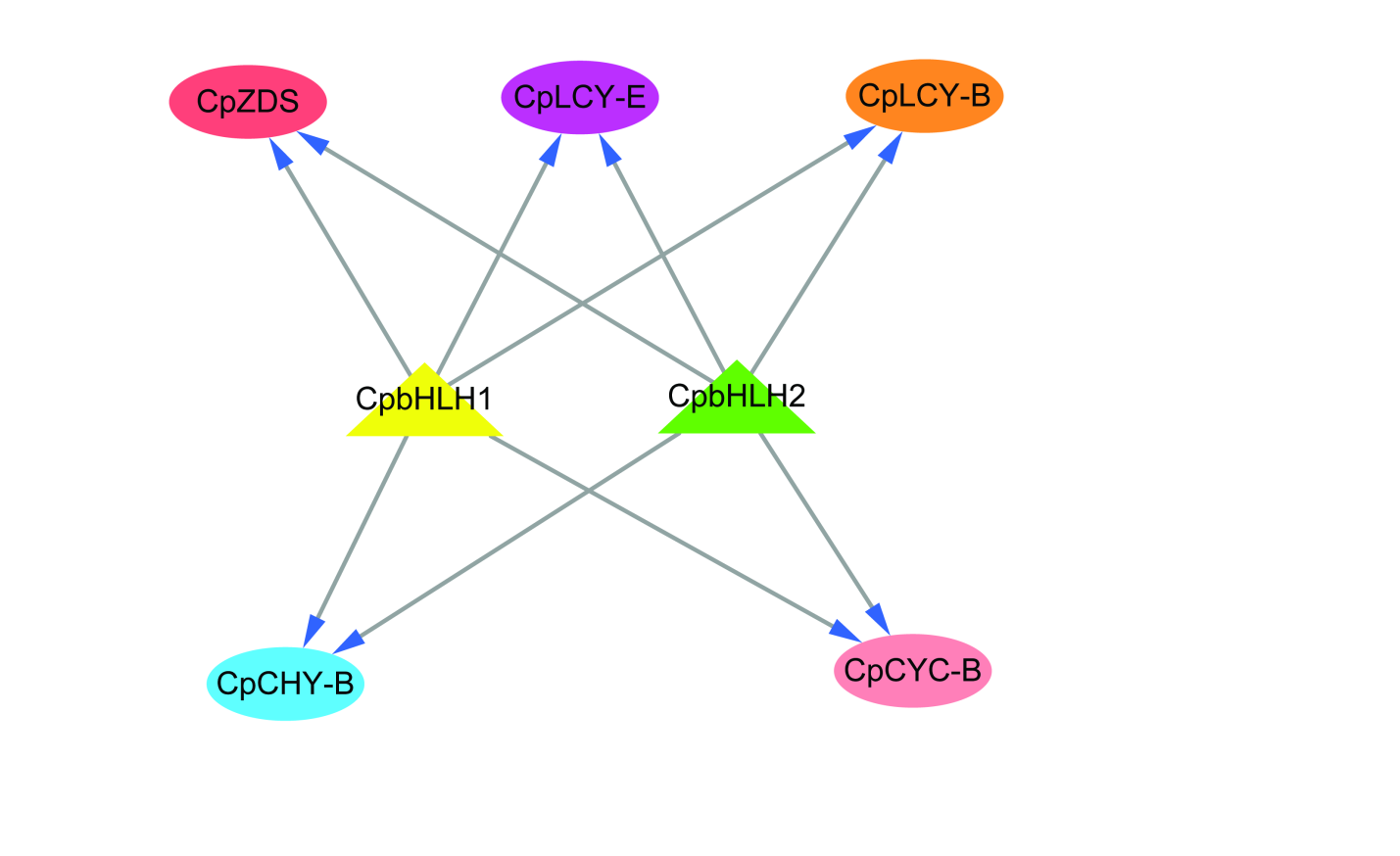


Figure S6. Co-expression pattern of *CpbHLH-1/2* transcription factors acting with targeting genes was shown.


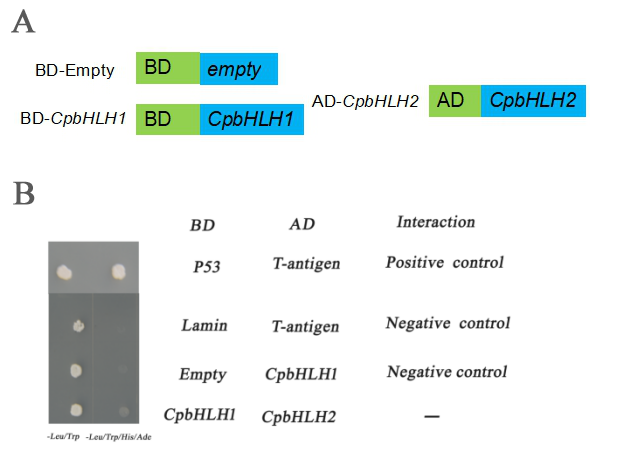


Fig.S7. Yeast two-hybrid system was tested between *CpbHLH1* and *CpbHLH2*. (A) Schemical pattern of yeast two hybrid interaction was shown; (B) Interaction was indicated by the ability of yeast cells to grow on a synthetic medium lacking tryptophan, Lucien, histidine and adenine(-Leu/Trp/His/Ade). Yeast cells transformed with pGBKT7-p53+pGADT7-largeT were used as positive controls, while those transformed with BD-empty+AD-*CpbHLH1*,pGADT7-LaminC+pGADT7-largeT were use as negative controls. The result (BD-*CpbHLH1*+AD-*CpbHLH2*) showed no interaction.
