# Supplementary material for: Papaya CpbHLH1/2 regulate carotenoid biosynthesis-related genes during papaya fruit ripening
Source: Hortic Res. 2019 Jun 22;6:80. doi: 10.1038/s41438-019-0162-2 (PMC6588581; doi:10.1038/s41438-019-0162-2)
Supplement: Supplementary file 2 — Supplementary Tables [file 41438_2019_162_MOESM2_ESM.docx]

**Table1. Primer sequence used for constructing various plasmids in this study.**

| **Assay** | **Primer sequence** | | **Restriction Site** |
| --- | --- | --- | --- |
| **Transfactors for yeast one hybride assay** | nNAC-594.1-up | GGAATTCCATATGGAATTCC ATGGATGATCAAGAAACAGTTGTTAAC | 55°，NDEL,BamH1,1122 |
|  | NAC-594.1-down | CGGGATCCCG TTAGTAGTCCCATAAACAATCTAAATCTGCAA |  |
|  | ERF-13.250-down | CGGGATCCCG CTATCTAACCAATAATTGCTCGCCAACTGG | 60°，ECOR1,BamH1,744 |
|  | ERF-13.250-up | GGAATTCC ATGGCCGTAGAGTATCACTTTCCTCA |  |
|  | ERF-131.83-up | GGAATTCC ATGGCTCCCAGGGAAAAGCTTC | 61°，ECOR1/XHO1,681 |
|  | ERF-131.83-down | CCGCTCGAGCGG TCAGGCCACTTCAGCTGCG |  |
|  | b-ZIP-244.3-up | GGAATTCC ATGGAGCAAAAACCGGCTCAG | 59°，ECOR1,BamH1 |
|  | b-ZIP-244.3-down | CGGGATCCCG CTAATTAGGCCAAATGCCTGATACGC |  |
|  | Bhlh-8.253-up | GGAATTCC ATGGAGATGAGGGATATAAAGCCCGT | 60°，1044bp，ECOR1,BamH1 |
|  | Bhlh-8.253-down | CGGGATCCCG TCACCGACAACTCTTCGAAGGATC |  |
|  | Bhlh-131.7-up | GGAATTCCATGGATATTGGATTTGGGTGGGATAGTG | 60°，1257bp，ECOR1,BamH1 |
|  | Bhlh-131.7-down | CGGGATCCCG TCAAATTTGTGTGGGGTAGTGCTG |  |
|  | Bzip-81.160-up | GGAATTCC ATGGAGGGTATTACAGAAACTGGTAAGAAGC | 60°，1227bp，ECOR1,BamH1 |
|  | Bzip-81.160-down | CGGGATCCCG TTATTCCCTGTCATGCATATTAGAATCGCCA |  |
|  | bZIP-9.75-up | GGAATTCC ATGGTGAAGCAGGGGATTCAGC | 60°，474bp，ECOR1,BamH1 |
|  | bZIP-9.75-down | CGGGATCCCG TTAAAACTCGAAGATCCCAGGGGATATAATCG |  |
| **Promoter cloning assay** | *CpCYC*1500pro-F | GTAGGTACCAGGCAATAGTCAGGGCTGTAATT | Kpn1 |
|  | *CpCYC*1000pro-F | GTAGGTACCTGAAAGCCTGAGAAGTTGCC | Kpn1 |
|  | *CpCYC*500pro-F | GTAGGTACCACACTAACCACCCAGGAGTAT | Kpn1 |
|  | *CpCYC*-d | TATGGCGCGCCAGATGTAAAAGGAGTAGGAAGAAGG | Asc1 |
| **Promoter cloning assay** | CpLCY1500pro-F | GTTGGTACCGGTATTAGGATTGAACATCAAC | Kpn1 |
|  | CpLCY1000pro-F | GTAGGTACCTAATTGCTCTGATTCTGACC | Kpn1 |
|  | CpLCY500pro-F | GTAGGTACCCTTTTCTCAGACTCAGAGG | Kpn1 |
|  | CpLCY-d | TATGGCGCGCCGGAATACCCAATATGAATTTAAC | Asc1 |
| **Yeast two hybride assay** | CpbHLH-1（BD）-up | CATATGGCCATGGAGGCCGAATTC  ATGGATATTGGATTTGGGTGGGATAGTG | Infusion;  60°，1257bp |
|  | CpbHLH-1（BD）-dn | GGCCGCTGCAGGTCGACGGATCCC  TCAAATTTGTGTGGGGTAGTGCTG |  |
|  | CpbHLH-2(AD)-for | GCCATGGAGGCCAGTGAATTC  ATGGAGATGAGGGATATAAAGCCCGT | infusion  60°，1044bp |
|  | CpbHLH-2(AD)-DN | TGCAGCTCGAGCTCGATGGATCCC  TCACCGACAACTCTTCGAAGGATC |  |
|  | CpbHLH-1（AD）-up | GCCATGGAGGCCAGTGAATTC  ATGGATATTGGATTTGGGTGGGATAGTG | Infusion;  60°，1257bp |
|  | CpbHLH-1（AD）-dn | TGCAGCTCGAGCTCGATGGATCCC  TCAAATTTGTGTGGGGTAGTGCTG |  |
|  | CpbHLH-2(BD)-for | CATATGGCCATGGAGGCCGAATTC  ATGGAGATGAGGGATATAAAGCCCGT | infusion  60°，1044bp |
|  | CpbHLH-2(BD)-DN | GGCCGCTGCAGGTCGACGGATCCC  TCACCGACAACTCTTCGAAGGATC |  |
| **Dual LUC assay** | PG62-bHLH-1(UP) | TGAGCTCCACCGCGGTGGCGGATGGATATTGGATTTGGGTGGGATAGTG |  |
|  | PG62-bHLH-1(DN) | GATAAGCTTGATATCGAATTCTCAAATTTGTGTGGGGTAGTGCTG |  |
|  | PG62-bHLH-2(UP) | TGAGCTCCACCGCGGTGGCGGATGGAGATGAGGGATATAAAGCCCGT |  |
|  | PG62-bHLH-2(DN) | GATAAGCTTGATATCGAATTCTCACCGACAACTCTTCGAAGGATC |  |
|  | P0800-CYC(UP) | CAGCCCGGGGGATCCACTAGTGTCATGTGTTCCCCTCCAAAACTG | 191bp,including-CACCTG-,--CACTGC |
|  | P0800-CYC(DN) | GTTTTTGGCGTCTTCCATGGAGGAAGAAGGTAGAGAGAGAACAGTCTGT |  |
|  | P0800-LCY(UP) | CAGCCCGGGGGATCCACTAGTCTCTCCTCTTGGTTCTAATTCTTTCCGAG | 216bp,including-CATTTG-,--CACTGC |
|  | P0800-LCY(DN) | GTTTTTGGCGTCTTCCATGGATGCAGGCAAAATCAAACTCAGGTT |  |

**Table S2 Primers sequence used for RT-qPCR**

| **Assay** | **Gene name** | **Primer sequence(F/R)(5’-3’)** |
| --- | --- | --- |
| qRT-PCR **primers for putative transcription factors** | Bzip81.160-RT-U | CCAAATCTGAGCTCAAAAAGATTATGGCTAAC |
|  | Bzip81.160-RT-D | GTACTTTTTGCTCCAATTCTGCAACAT |
|  | Bzip9.75-RT-U | CGTGATTACGGACGAGAAGAGAAG |
|  | Bzip9.75-RT-D | CAAACCTCTTGTTTCCCTCCTGTAT |
|  | NAC594.1-RT-U | CTGCCAGAAAGATAGAAAGTATCCAACCG |
|  | NAC594.1-RT-D | GGAGCTCTCCCTCTGTAAAAAACAAGAG |
|  | ERF131.83-RT-U | AGAAGACAAGAGTCTGGCTCGG |
|  | ERF131.83-RT-D | ATTGTTCGCACCACCGTTATTGT |
|  | Bzip244.3-RT-U | ACAGAAAGCCTGCTATGGTCTCAAA |
|  | Bzip244.3-RT-D | ACTGGCAGCTCCTCTGACTTT |
|  | ERF13.250-RT-U | GCCAAGAGGAAGGCATTATCGAGG |
|  | ERF13.250-RT-D | CGCTCGGTCATACGCGAAAG |
|  | CpbHLH1-RT-U | CTCATCGTCGGTATCGTCGTGCAT |
|  | CpbHLH1-RT-D | GCTTCTCCACCATCTCCAACCCAAGATTC |
|  | CpbHLH2-RT-U | CACTCATCTTCTTCCTCATCCGCTCGTAACA |
|  | CpbHLH2-RT-D | GCTGAGGTGTAGCTCTGGAAGTTGAAGTTG |
| **qRT-PCR primers for carotenogenes** | CpCHY-b-RT-U | AGTTCCTGCTATCGCTCTTC |
|  | CpCHY-b-RT-D | AATAAGGCACGTTGGCGATG |
|  | CpLCY-e-RT-U | TGATTCTGCAGCCAAAGCTA |
|  | CpLCY-e-RT-D | ATATAAAGGCCGGTGGCTCT |
|  | CpZDS-RT-U | CTTCTTTACACACCAGATGCCGA |
|  | CpZDS-RT-D | GCCTTGTGATGAAGGGAACAAG |
|  | CpTBP1-RT-U | GGTAGTAGTAGTTAGGTATGTG |
|  | CpTBP1-RT-D | GGCAATCTGGTCTCACTT |
|  | *CpCYC*-b-RT-U | GGTCGTTGATGCAAGTGGAT |
|  | *CpCYC*-b-RT-D | GGTCTGCTAACCAGAGAAGT |
|  | CpPDS-RT-U | TGAAGTTTGCGATTGGGCTTCTGC |
|  | CpPDS-RT-D | ATGCCCTGCTTTCTCATCCACTCT |
|  | CpLCY-B-RT-U | TGGAAGGGTTAACAGGAAGCAGCTGAAG |
|  | CpLCY-B-RT-D | CAACAAAGACTTTGATTCCTCGTGAATGGC |

**Table S3 Primers sequence used for Phis2 vectors**

| **Assay** | **Binding sites name** | **Primer sequence(F/R)(5’-3’)** |
| --- | --- | --- |
| **Phis2 vectors for**  **Yeast one hybrid** | bHLH -up | aattcCACTGCCACTGCCACTGCCACTGCCACTGCgagct |
|  | bHLH -dw | gGCAGTGGCAG TGGCAGTGGC AGTGGCAGTGc |
|  | NAC-up | aattcCGTGCGTGCGTGCGTGCGTGgagct |
|  | NAC-dw | gCACGCACGCA CGCACGCACGc |
|  | ERF-up | aattcGCCGGCCGGCCGGCCGGCCGgagct |
|  | ERF-dw | gCGGCCGGCCG GCCGGCCGGCc |
|  | bZIP-up | aattcAGACGTAGACGTAGACGTAGACGTAGACGTgagct |
|  | bZIP-dw | gACGTCTACGT CTACGTCTAC GTCTACGTCTc |
|  | _bHLH mutant-up | aattcCATCACCATCACCATCACCATCACCATCACgagct |
|  | _bHLH mutant-dw | gGTGATGGTGA TGGTGATGGT GATGGTGATGc |
|  | NAC mutant-up | aattcCACGCACGCACGCACGCACGgagct |
|  | NAC mutant-dw | gCGTGCGTGCG TGCGTGCGTGc |
|  | ERF mutant-up | aattcGTTGGTTGGTTGGTTGGTTGgagct |
|  | ERF mutant-dw | gCAACCAACCA ACCAACCAACc |
|  | bZIP mutant-up | aattcAAGTGTAAGTGTAAGTGTAAGTGTAAGTGTgagct |
|  | bZIP mutant-dw | gACACTTACAC TTACACTTAC ACTTACACTTc |
|  | NAC2- up | aattcCGTACGTACGTACGTACGTAgagct |
|  | NAC2-dw | gTACGTACGTACGTACGTACGc |
|  | NAC2 mutant-up | aattcCACACACACACACACACACAgagct |
|  | NAC2 mutant-dw | gTGTGTGTGTGTGTGTGTGTGc |
|  | Seg1-UP(2,3,4) | CGGAATTCCG GTTTGGTAACAAAGAAGGATAGTATCG |
|  | Seg1-DN(2,3,4) | CGAGCTCG CACAAATGTGAGTAGAAGAATAGTG |
|  | seg2-UP(1) | CGGAATTCCGCTGCAAAATTTTTGTCGTCATCTT |
|  | seg2-DN(1) | CGAGCTCG GACCACAGAGAGACAGGGA |
|  | seg3-UP(2a,3,4) | CGGAATTCCGCTTAGGAGGCCGAGATAAATGC |
|  | seg3-DN(2a,3,4) | CGAGCTCGGAATTTCATAATCTTCTTCACGAGTGCTG |

**Sequence1.**Nucleotide sequences analysis of the carotenogenes promoters. The bHLH DNA-binding site (PBE-box) core sequence(-CANNTG-)were underlined, the target sequence used in yeast-one hybrid assay is indicated in bold. In promoters of *CpCYC-B* and *CpLCY-B*, transcription family bindng-sites were marked by number, for example (1)bHLH, (2)NAC, (3)AP2/ERF, (4) Bzip. Three segment sequences and two segment sequences were taken from *CpCYC-B* and *CpLCY* –B promoters respectively, shown in above sequence.

**Sequence1**

***CpPDS2 (evm.model.supercontig_157.3) promoter***

ATCGGAATATTCTCGTCTCCCATCAAAAAGAGAAAAAGAAAGAAACGAAACGAAAAGAAAAGAAAAAAAGTCCCCCAAAGCCAAGGGATCAGAAGAGCGCTACAATCCAAAATTACATGGGAAGCTCAAGTTTGGCCAGTGCACTTAGTTAATGCCTATCACCAGCTTCCACATATGTTATCAACAACAAGCCCACTTTCCGTTTTGTGTTATTGACAAAATCAACGTGTTTTCTTGAGATTGAGCTCTCTTCTGGTTTTTCTTCTGGGTGCGGTTGAAGGGAGCGTTGTCGATTTTACTGGTATTAGCATTTTGGTTTGTTGTTAATGATATTTCTCATTTTGAGCTCGTCAGAGCAAAGATAATATACAGAATTTTCAGCTTTCTTAGTTTCACGAAACCAGATAGACATTACCCAGAATCAGAAAAGGAGAAAACTAGTTGGAAATTATTCTGATTTTTTTTTTTT**CGGGTTTGTTGTTCTGAACTCTGTGTCAGAAGTTTCAGTCTCTATTATGGAGGAGTTAATAATTGCATGCCCATTTCGATTTTTACAGCAGCTCTAACTTCTATTATCTTTTTGTCCATGGAAAGCTTTTTGACTCGGAAGTTGCTTTCAAACTGTTCGATAAAAGCAAAGTTTGGCAACTGCTGAAATG**

***CpPDS4* (evm.model.supercontig_6.114) promoter**

GTATCCAACCAAGCACGCCACACAGAAGTGCTATTAGCTGTTCCCAATAATTATATCATTTGGGTTTTAGCTTTCAAAAATAAAAAATAAAAAACTCTGACAGTTGACTAAAAAAAATTGCGGCTACCTAACAGAAAAAGACATGAGTGGAAAGTAAGAACCTTAAACGGGTTTCGGTTTCTGGTCGGGGCCATGTCTCCTCTGCTACTCGGTCGGAGCTCCGCTTTGTTCGGAGAAAGATGCTGTGTCGTGTAGATGGGCTGTGCATATTTATATACTAATACGGTATTTACACATGATA**ATCGGCCTTTTCATTTTTTGACATCTACTGTTAAGTGTGCTGCGGTGCTGACTTTTATGCTAGTTGGCCAATGGTAGGATGACACGTGGTTATTACCACAATTATCATGCGCATAACTGTAATATGTTTCACCTTACATAACTAACGTACTTTTAAAATATTTTAATCTCGTATT**AAAAAAATATATAATTTTTAAATTTTATATAACAATTTAAATCATACTATTAATAATTTTAACTTAAATTTTTTAATACAAATTATTTGAATCCAAAAAATTATTAAATTAATAATATTTAAATTCAAAACATTATAAATAATATTAAACCGTGTGTTAATTCAGTAATTTTAATTAATATAATTTTAAATTTTATAAATTTTTAATATATAATTTATGATTATATAATGTGAGTAAATTAAGTATTTATGGAGATATTATAATATTTTAAAATTATAATAAAAAATATATAATTATTAAGTTGTATATAAAAACTTAAACCATATGGTACAAATAACTTAGAATTTAGACTCTTAAAATTTAGTGGTATGGATTAAAAAAATTATTGAATTAATTTATGTTACCATTTTACTTTTCTCATTTTTTTAAACAATAATTATTGATAAATATTTAAACTCTCAAGGGTCCCACCTCAGCTACCTTAATTCCGTCATCCTGATTCCTGCAAAAGAAAGAAAGCAAAGAAAATGAAAATGCTGACGGTGACACCCTCTGTCACTATGAAACGATACTCCATTAAAGGGCCAGTCAATGAGCTCGGCGTCGATTACAGACCAACAAAGCTTCCTTAGGTTCAGAAATG

***CpZDS* (evm.model.supercontig_117.67) promoter**

CGTTTGAATATGTCATGACTCGTTGGATAAAATTCAATTTAGTCAACACATTAATAAGTTAAGGTTTTGATTTTATTAAATCAAATATTATAAACTGTTCATTTATATTCGTAGAGTGATAAATTTTATATCATTGATAAATTTCAAATTCATGACATTTAAAAAAAATATAAAATTTTTAATAATTAGATCACCCTACTATGTGTAGTAAAAAAATATCTCCTTTCGTACCTAAAAATTTACCCTTAACAACTTTAATTAAAAACATATAAGCACTTCATCATACTCACAAAAAAATTTTGATTGTTGGGGTGAAATTTGAGTGGAGGGGTTTTCCTAAGATTGAATTTAACATAAATTTCTAGCATTTTTTCCTCAAATTTTCAAAAAAATTTAAATGATCAAACTTTTAGAATATATTTAAAATAGATGTCGAGGGGATATTTTTTTTGACGAACGAGAGTTGTACCCTTCGTTGTTTATTATTTTTATAGAATTTATTTAGTAATTAATACTAAATTTAAGTAGAAATGTTAGAAATTCGATTAGAAATAGTAAAACAATAGTTTTGTATAGTTTAATATAAATTTTTAAATTTATTGTGGTTAATAAATATTTTAATTTATACTTAAAAACTCTCTTTAAACTTATTAAATATAAATTTTTAAATAAATTTTATAAAAATACAAAAGATGGTGATAATAAATCAAAAAAAAAAAAACAGAAAACTCTTTGAATACCATTTTTTTTTTCTTTTTTTAGTAAATAGAAACTCC

TTTAGGATACCTCTTTAGAATACCCTTTGGTTCTTTAGGAATGGTTGGTATGCATTAAGCCATTATAGATAGTGGCAATG**AAATGGGTCACTAACATTTCTTGTCCACTTTCAGTTCTTAGTGCAGAAAATTGTATGTTAGATAAAAATTAAAATAATCCAATCTTGTTGCCAACTGCAATAACCTCCAAAGGCAAATCCAAATCCAAAGCTCTTTCCTTTTTTTTATTTTTTAAAGAAAACAAGAGGAATCTCAAGAGCCTTGTTTGCTGAC**AAATCTCAATCTCTCAGCATCTCTTTCTCTCTAGATACCTTCTCCTTGACCCTTTTCTTCCAAAATTTTCATTTTTACTAATGATACAATCAAGTCCTTCGGTTTTGACACTCGGGTTTTTCTTGGATTGTTAGATAGAGTGAAAAAGAGCTCTACTTTTCTCTGAAATTTGATTTGGTTTTTAGTGGGTATTTAGTTTTTGTGTGTCTATG

***CpLCY-E* (evm.model.supercontig_28.134) promoter**

GATGTTGACGAGGAGGACGTGATGAAAGCTTTCTTTAATTGGATTTTTTTTTTTTTTTTTTTTTTTTGTAATCTTATTAATAATCAGTGCACTTTATTTTATAAATGAAATAAATTTGAACATTATCGAAAGAAAGATTACCCATTATTTAATAATTAGTACATGTTTTATTGAAGAGGTAATGAGACTTATTTTTTGGATTTTCTTATCATAAATGTTTTCGTGAAATCAAATTTTTTATAAGTTTTTGTAGTATCATTAATGTATTTATAAAATGA**AAACTACGTGGGTTACGGGCGAGTCCATTAAGTCCTCAAGCCCGGAAAAAGACTTGTTTTGCGGCACAGATCCATGATTCACGTCAAAACGCCCAGTTTCATTTGCGATAGGAAGTAACTTTTTCAAACAGGATAGCCAATCCAAAACGTCCACGTGGCAGCATGAGGATGGCGGTTTAAACAGCT**CAGCAAATCATAAAGACTAGTGGCCTTCCCTTCTCCTATCTGTTGCTGAGAAACAGAGACTTGAAGAAACAACAAGACGAAGAAAAAAGAGGGAGAAGAATTTAGATTTTTTCAGCGCATTATTTTCCTCCCTCCGCAAAAAGTCTTCAATCACAGTAATGACGTAAAAACATTTCTCATTTGAAAGTTTCTGTCCAAGGAAACAAGAGAAAAAAGAGTAGAATATG

***CpCHY-B* (evm.model.supercontig_107.106) promoter**

CCGTGACGGTGAGATAGTGTGATGTTTTTACTGGGCTGGTGTAGCTCATTCCTTATTTAATCATTTCAGATTAGGAAGACATTGTTCCCGAGCCCGTTCCGCAGGAAGACGGCGAAGGGGACGCCTGAAGACTCCGTAGTCATATTTATAGCTTTTATTTTCGAGTTTCTCTTTTAGTTAATTTGTTTTTTTATACTTTGAGCACGTTTTTGGTTTTAGTATAAGGGATGTTTAATCAAAATTTTTATTACGCTTCCGCATTTGACCTTTTAATACACATATTTCATTTTAAATTTCCGCGGCCGTTACATTGAAAACGAAAGGGTAGGCGGATAGGAGTTGAAATTGGTCATACCATTTTAATAATTCACATTTCTATTTTCTCGTTATAAAAAAATTATAATTAAATTATAATTAATTTAATTCGTGTGTATTATCCATTTAATTGTATGGTAATATTATGTAATTTTTTAATTAAATTTTTTTTCATTTATCATTTTAG**AATTATTTAAATTATTATCCACTTTATTAGTAGTTTTAATTACAAATTATAGTAATACGGGGGGTGGCGCCAATAATTTCAGTGGCATCAACTGTAATTTAAGGCAATATCGATGTCAAATTTTTAAGACCATGCCTAAGTCTATTTCTGAGAGACGGTTAGTTTTCATCTTAAAAAGGAGTCAAGACTGATC**GAGAACGAAGATGTGGTCTCTGTGCATTTAACTCTCCACTCCTCCAAAAACAAAAAAAAACTCTCTCCTTCAGTA

GGCTCCAAACCTAGGTTAGAGACCATACCCGGCCCCCACCGTCCATCTCTATCTCTCTGCCTGTCGATAATTCTTGCGTTTGTTTTGTGTGTTTTCTGTGTGGGTTTTTGTTGGGTAGAAACCTTTCGTCACAGTTCTCCTTCAAGCTCCTTCTATG

***CpCYC-B***

TTTGAAATTCTGCTTCCTGTAATTCCATGCAAATGGTACTTTGCAATTCGCTGTGGTTGCTCTCAGTTCTCTTATAGCTATAGGACAATATCTTAAATGC**ATATTAATCTTAGTAAAACCAATTAATTTGTTTTGTTTGGTAACAAAGAAGGATAGTATCGTACAAAAAGAATAAGAAAAGATGTGCAACAATTTAAACTCTTTTTTAAACCCCGATCGTAATACAAACATTACCGTCGGTCGAAATATATCTTGTACCTTAGGAGGCCG(3)AGATAAATGCAATGAGAACGAAGACGT(4)TGAGCTTGG**TGATTATAAGCTTAAAATT**TCACTATTCTTCTACTCACATTTGTGTTTATAAGTGTTCTTCTCAAACACTAACCACCCAGGAGTATTTCTAACACCTCTTACACAGCACTCGTG(2)AAGAAGATTATGAAATTCTTAAATTTTTTCTTTATTAAATAGTGATTTCAGAATTTTGTTATGCATG(2)TAAAACATTTTTAAGTAAAATTTTTGAAATAATTT**TGTAGCTTTTAATTCAAAAATAAAAAAATCCTAAATTTTATAAAAATAATTACTTTATAAATATATTATAATTTTATAATATTTAATTTAATATAATTTAATGTAAAGAGTAGATTT**GTCATGTGTTCCCCTCCAAAACTGCAAAATTTTTGTCGTCATCTTATATTTGCACACTGCCAGAACAACCTCCATTTTTGAAGTTGCGCCAGAAGGCATCCTTCTTTCCACTCCACCTG(1)CGATCCTTTTTTCTGTCTTTATCTCCCGCTTCATTTCTTCTCTACAGACTGTTCTCTCTCTACCTTCTTCC**TACTCCTTTTACATCTCTCCCTGTCTCTCTGTGGTC

***CpLCY-B***

TTTTACATTTTCATTTTTAGAATTTCTAATTAAATTTTTTATTTATTATATATATTTTTAATTGTATAAAAATATTAGAAATTATTTAACTAAATGAAAGGTGGGTATGGGTATTTATTTTTCGATGGTGGGAATTCCCTCCAATATTAAATTTATTTATTTATTTATTTATTTTTATCAATATCTTTTCTCAGACTCAGAGGGTCCCCCAACGCCTTCAGCTTGTCGTCAAGAAGTGGCCACCCAACTATCGTCACCTCACTTCGAAGGCTCCAACAAAATCTCATGGCTTCTCTTTATCTTCTCCTTCACTTTTCCTATCGACCTGGTAAGTTATCTTGTAGCAAT**CTCTCCTCTTGGTTCTAATTCTTTCCGAGTTCCATGTAATCAAAATATATTTATTTTTTTGTAATCTTGGAGTTAGTGAATACCCAGTTCAGTATCTCTTCAATTCACTGCCTTTTTTGCTGTAAATATTTCTATATAGGTGATTGTAGAGACCCACTTTATGAATTACATTTG(1)T*CGAGATTTTATTAGGAAAACCTGAGTTTGATTTTGCCTGCAATTTAATTTCAGCTGAGAACTAGACAGACTCTACAAAGCCG(3)AGTTTGTGTTCACAGGAATTTTTTGGTAACCCCAATTTGAGTTTTAGGGGTTAAATTCATATTGGGTATTCC***
